# Toward combining qualitative race-specific and quantitative race-nonspecific disease resistance by genomic selection

**DOI:** 10.1007/s00122-023-04312-2

**Published:** 2023-03-23

**Authors:** Sebastian Michel, Franziska Löschenberger, Christian Ametz, Hermann Bürstmayr

**Affiliations:** 1 Department of Agrobiotechnology, IFA-Tulln, University of Natural Resources and Life Sciences Vienna, Konrad-Lorenz-Str. 20, 3430 Tulln, Austria; 2 Saatzucht Donau GesmbH & CoKG, Saatzuchtstrasse 11, 2301 Probstdorf, Austria

## Abstract

**Key message:**

**A novel genomic selection strategy offers the unique opportunity to develop qualitative race-specific resistant varieties that possess high levels of the more durable quantitative race-nonspecific resistance in their genetic background.**

**Abstract:**

Race-specific qualitative resistance genes (R-genes) are conferring complete resistance in many pathosystems, but are frequently overcome by new virulent pathogen races. Once the deployed R-genes are overcome, a wide variation of quantitative disease resistance (QDR) can be observed in a set of previously race-specific, i.e., completely resistant genotypes—a phenomenon known as “vertifolia effect.” This race-nonspecific QDR is considered to be more durable in the long term, but provides merely a partial protection against pathogens. This simulation study aimed to detangle race-specific R-gene-mediated resistance of pending selection candidates and the QDR in their genetic background by employing different genomic selection strategies. True breeding values that reflected performance data for rust resistance in wheat were simulated, and used in a recurrent genomic selection based on several prediction models and training population designs. Using training populations that were devoid of race-specific R-genes was thereby pivotal for an efficient improvement of QDR in the long term. Marker-assisted preselection for the presence of R-genes followed by a genomic prediction for accumulating the many small to medium effect loci underlying QDR in the genetic background of race-specific resistant genotypes appeared furthermore to be a promising approach to select simultaneously for both types of resistance. The practical application of such a knowledge-driven genomic breeding strategy offers the opportunity to develop varieties with multiple layers of resistance, which have the potential to prevent intolerable crop losses under epidemic situations by displaying a high level of QDR even when race-specific R-genes have been overcome by evolving pathogen populations.

**Supplementary Information:**

The online version contains supplementary material available at 10.1007/s00122-023-04312-2.

## Introduction

Developing cultivars with race-specific qualitative resistance genes (R-genes) by either classical phenotypic or marker-assisted selection to control obligate biotroph pathogens is a convenient option for plant breeders, since these genes provide complete protection against a particular pathogen complex and are relatively easy to manipulate (Poland et al. 2009). The underlying gene-for-gene relationship, which states that for each gene controlling resistance in the host there is a corresponding gene controlling virulence in the pathogen (Flor 1955, 1971), is one of the most important concepts for understanding plant pathosystems to this day (Kaur et al. 2021). Deploying such R-genes can furthermore be considered as a suitable short-term strategy to offer farmers highly resistant varieties and limit the application of plant protection agents like fungicides. However, the high evolutionary dynamics and the regular occurrence of new virulent races for many pathogens like the wheat rusts (Ellis et al. 2014; Hovmøller et al. 2016; Zetzsche et al. 2019) may render previously resistant varieties highly susceptible after a short period of cultivation (Pink 2002; Stuthman et al. 2007; Sørensen et al. 2014; Laidig et al. 2021) and lead to the frequently observed boom-and-bust cycles (McDonald 2010). One such event occurred when the stem rust race “Ug99” overcame the widely deployed resistance gene *Sr31* in wheat, leading to severe epidemics in Eastern Africa in 2004 (Singh et al. 2015). Another prominent example is the occurrence of the new and highly virulent stripe rust race "Warrior," which spread rapidly across the European continent in 2011 and resulted in the collapse of the formerly effective race-specific resistance in wheat that was conferred by several *Yr* genes (Hovmøller et al. 2016).

Race-nonspecific quantitative disease resistance (QDR) is on the other hand considered to be more durable than this race-specific R-gene-mediated resistance, but provides merely a partial protection against pathogen attacks (Cowger and Brown 2019). The mechanisms underlying QDR are manifold and involve genes regulating plant morphological characteristics, components of signal transduction systems, and antimicrobial compounds such as phytoalexins (Poland et al. 2009). The genetic architecture of this QDR can furthermore be highly complex as for example many small to medium effect quantitative trait loci are influencing the quantitative disease resistance against stem, leaf, and stripe rust in hexaploid wheat (Pal et al. 2022). Furthermore, a mixture of both race-specific and race-nonspecific resistant genotypes for obligate biotroph diseases like the wheat rusts can oftentimes be found in the germplasm of applied breeding programs. An especially interesting case occurs when the magnitude of QDR in the genetic background of race-specific resistant genotypes is phenotypically masked by the presence of the according R-genes that are conferring a complete protection against an infection with the pathogen. Once the deployed R-genes are overcome by new pathogen races, a wide continuous variation from resistance to susceptibility can then be observed in the set of previously race-specific, i.e., completely resistant genotypes—a phenomenon that has become known as the “vertifolia effect” (van der Plank 1963). The genetic foreground that can be observed of such genotypes in the field and a given year of selection is thus similar, i.e., all genotypes appear to be completely resistant, while the occurrence of new virulent pathogen races in subsequent years can reveal large differences of disease resistance among these genotypes due to different levels of QDR in the genetic background (Cowger and Brown 2019; Laidig et al. 2021). Although this genetic background cannot be phenotypically observed in a given year of selection, it might be feasible to detangle the race-specific R-gene-mediated resistance in the genetic foreground of young generation selection candidates and the QDR in their genetic background by employing high-throughput genotyping technologies (Poland and Rutkoski 2016).

The cost-efficient availability of such high-throughput genotyping technologies and methods like genomic prediction with genome-wide distributed molecular markers has revolutionized the breeding for disease resistance in recent years (Miedaner et al. 2020; Miedaner and Juroszek 2021). The basic idea of genomic prediction is given by fitting a prediction model with a phenotyped and genotyped training population in order to obtain genomic estimated breeding values of selection candidates for which only genotypic information is available (Meuwissen et al. 2001). Several studies concerning the usage of these genomic estimated breeding values to support selection decisions reported promising results in multiple pathosystems (Rutkoski et al. 2015a; Jiang et al. 2017; Stich and Van Inghelandt 2018; Herter et al. 2019a, b; Beukert et al. 2020b). Factors that appeared thereby pivotal for a successful implementation of genomic prediction are a high enough marker density (Stich and Van Inghelandt 2018), training population size (Rutkoski et al. 2015a; Herter et al. 2019b) as well as a close genetic relationship between the training population and the selection candidates (Jiang et al. 2017). Modeling genotype-by-environment interaction, for example in the genomic prediction of stripe and leaf rust in wheat, has furthermore shown large merit for implementing sparse testing strategies and optimizing resource allocations for disease resistance screening by breeders in a given year of selection (Semagn et al. 2022a, b). The genomic prediction for disease resistance across different years and cohorts can on the other hand be a very challenging task in the light of the race dynamics of pathogens such as stripe and leaf rust (Beukert et al. 2020b), as training populations in applied breeding programs usually consist of individuals that were phenotyped in years preceding a given year of (genomic) selection of nonphenotyped young generation individuals (Robertsen et al. 2019). This can invoke a strong discrepancy between the training population and selection candidates in the genomic prediction of disease resistance, since the phenotypic data of the training population does not reflect the current situation of the selection candidates if new virulent pathogen races occur in a given year of selection. Despite the presence of strong race dynamics, few genomic prediction studies distinguishing between race-nonspecific QDR and R-gene-mediated race-specific resistance for the wheat rusts (Rutkoski et al. 2011, 2015b; Juliana et al. 2017; Azizinia et al. 2020). Focusing exclusively on the genetic improvement of QDR by using training and selection populations that were devoid of race-specific R-genes resulted thereby in some genetic gain for stem rust resistance in wheat (Rutkoski et al. 2015b), while high prediction accuracies were observed in the presence of race-specific R-genes if they were effective in both the training population and the population of selection candidates (Juliana et al. 2017; Azizinia et al. 2020). However, the effect of the above-mentioned discrepancy between the training population and selection candidates when race-specific R-genes are overcome by the pathogen as well as the associated “vertifolia effect” has not been investigated yet in the genomic prediction framework. Hence, true breeding values were simulated in this study to reflect performance data for quantitative and qualitative rust resistance in wheat, which makes it possible to distinguish between the genetic foreground and background of race-specific resistant genotypes with respect to their QDR and R-gene-mediated resistance. Using these simulated disease resistance data, the aim of this simulation study was accordingly to compare the effect of different prediction models and training population designs on the genetic foreground and background of the selection candidates during a recurrent genomic selection.

## Materials and methods

### Plant material and genotypic data

A panel of 1628 genotypes comprised F_4:6_, F_5:7_ or double haploid breeding lines developed in the applied winter wheat breeding program of Saatzucht Donau GesmbH & CoKG in Austria was analyzed in this study. All lines were genotyped with the DArTcap targeted genotyping-by-sequencing approach (Diversity Arrays Technology Pty Ltd 2020). Alleles of the SNP markers were coded as “−1” for homozygous minor, “ + 1” for homozygous major, and “0” for heterozygous. SNP markers with more than 10% missing data were filtered out as were SNP markers with a minor allele frequency smaller than 5% and a heterozygosity larger than 10%. This resulted in a final markers dataset of 1138 markers for further investigating the possibility to combine race-specific qualitative, i.e., R-gene-mediated resistance and race-nonspecific quantitative disease resistance (QDR) in a simulation study.

### Simulation and genomic prediction models

**Fig. 1:**
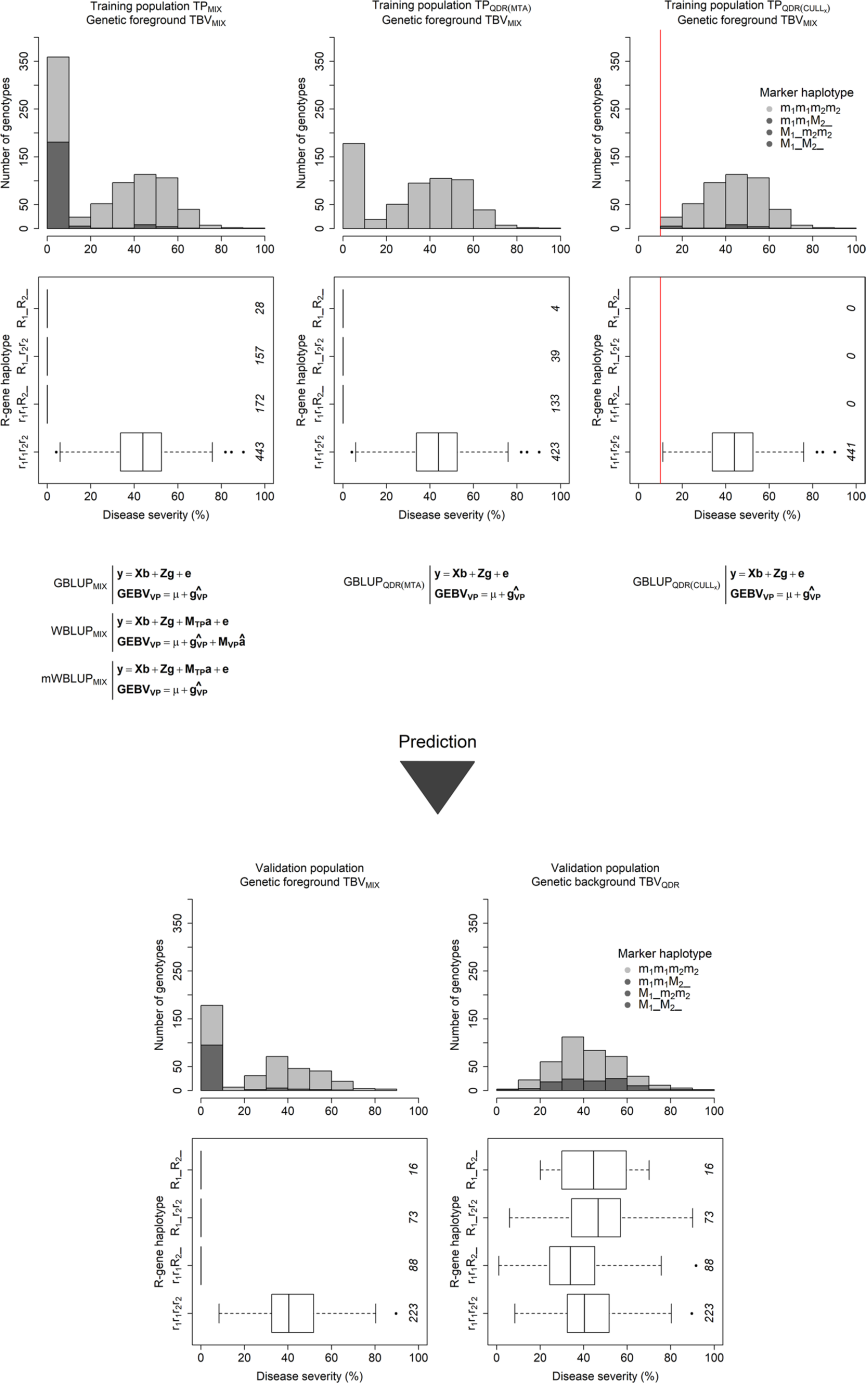
Example of one replication in the simulations, showing the variation of the simulated true breeding values that aimed to reflect scorings for rust resistance in wheat, their haplotype at the two simulated dominant race-specific R-genes $R_{1}$ and $R_{2}$ (number of lines in italics), and the haplotype at the linked SNP markers $M_{1}$ and $M_{2}$ that were identified by genome-wide association mapping in the training population. The baseline training population consisted of a mixture of race-specific and race-nonspecific resistant lines (${\text{TP}}_{{{\text{MIX}}}}$), which was used to derive genomic estimated breeding values by a basic genomic based linear unbiased prediction model (${\text{GBLUP}}_{{{\text{MIX}}}}$) or a prediction model that additionally included the linked SNP markers as fixed effects, which were either *upweighted* (${\text{WBLUP}}_{{{\text{MIX}}}}$) or *unweighted* (${\text{mWBLUP}}_{{{\text{MIX}}}}$) in the computation of the genomic estimated breeding values for the validation population (${\text{GEBV}}_{{{\text{VP}}}}$). Additionally, genomic based linear unbiased prediction models were fitted with a reduced training population that preferably contained only race-nonspecific quantitative resistant lines (${\text{TP}}_{{{\text{QDR}}\left( {{\text{MTA}}} \right)}}$) by entering solely lines that carried the susceptible allele at the linked SNP markers (${\text{GBLUP}}_{{{\text{QDR}}\left( {{\text{MTA}}} \right)}}$). Further training populations, which also contained preferably race-nonspecific quantitatively resistant genotypes, were derived by culling lines that had a true breeding value smaller than *x* = 10%, *x* = 20%, *x* = 30%, *x* = 40%, or *x* = 50% disease severity (${\text{TP}}_{{{\text{QDR}}\left( {{\text{CULL}}_{{\text{x}}} } \right)}}$) before fitting genomic based linear unbiased prediction models (${\text{GBLUP}}_{{{\text{QDR}}\left( {{\text{CULL}}_{{\text{x}}} } \right)}}$). It was assumed that the race-specific R-genes are still effective in the entire training population when using the different model-by-training population combinations were subsequently used to predict the simulated true breeding values for the genetic foreground (bottom left) of lines in the validation population, comprising both race-specific and race-nonspecific disease resistance, i.e., when race-specific R-genes were considered to be effective in the validation populations (${\text{TBV}}_{{{\text{MIX}}}}$), as well as their genetic background (bottom right), comprising solely the race-nonspecific quantitative disease resistance, i.e., when race-specific R-genes would not be effective anymore in the validation populations (${\text{TBV}}_{{{\text{QDR}}}}$)

The 1628 genotypes were for the purpose of the employed simulation layout 50 times divided into a randomly sampled training population of 800 and a validation population of 400 genotypes (Fig. 1). $N_{{{\text{QTL}}}} = 80$ marker loci were randomly sampled as causal variants conferring quantitative disease resistance and another randomly sampled set of $N_{{{\text{SNP}}}} = 800$ markers served as linked loci for fitting prediction models and conducting genome-wide association mapping. QTL effects $\alpha$ were randomly sampled from an identical and independent normal distribution with $\alpha \sim N\left( {0,1} \right)$ and used to compute true breeding values of all lines in the training and validation population by:

1 $${\text{TBV}}_{{{\text{QDR}}}} = Q\alpha$$

where $Q$ is the marker matrix of the genotypes in the training and validation population, $\alpha$ is the vector of effects of the causal loci, and ${\mathbf{TBV}}_{{{\text{QDR}}}}$ is the vector of simulated true breeding values for the race-nonspecific quantitative disease resistance that were transformed to a 0–100% scale to reflect scorings for rust resistance in wheat. ${\mathbf{TBV}}_{{{\text{QDR}}}}$ represented thus a genetic architecture where many minor to medium effect loci are influencing the disease resistance in a quantitative manner, while race-specific R-genes that provide a complete protection against an infection with the pathogen are absent or have been overcome by the pathogen.

Two additional markers with a frequency of the “ − 1” allele of 0.2–0.3, 0.3–0.4, 0.4–0.5, 0.5–0.6, 0.6–0.7, and 0.7–0.8 were subsequently sampled to represent the causal variants $\kappa$ and $\tau$ of two such dominant race-specific R-genes $R_{1}$ and $R_{2}$ in order to simulate a second genetic architecture that reflects the presence of an effective R-gene-mediated resistance. Both of these markers were sampled from different chromosomes based on a genetic map made available by the genotyping service provider (Diversity Arrays Technology Pty Ltd 2021) and used to simulate the according true breeding values:

2 $${\text{TBV}}_{{{\text{MIX}}_{i} }} = \left\{ {\begin{array}{*{20}l} {0{ },\quad {\text{if }}\kappa_{i} = - 1{\text{ or }}\tau_{i} = - 1} \hfill \\ {0{ },\quad {\text{if }}\kappa_{i} = 0{\text{ or }}\tau_{i} = 0} \hfill \\ {{\text{TBV}}_{{{\text{QDR}}_{i} }} ,\quad {\text{otherwise}}} \hfill \\ \end{array} } \right.$$

i.e., if the *i*th genotype carried the “ − 1” or “0” allele at the loci $\kappa$ and/or $\tau$ it was considered to be completely resistant due to the R-genes. Lines carrying the “ + 1” allele at both loci were on the other hand considered to lack the resistance allele at both race-specific R-genes and were thus regarded as being only partially resistant due to the combination of the many small to medium effect loci underlying race-nonspecific quantitative disease resistance. ${\mathbf{TBV}}_{{{\text{MIX}}}}$ represented thus a genetic architecture where many minor to medium effect loci are influencing the disease resistance in a quantitative manner, while race-specific R-genes that provide a complete protection against an infection with the pathogen are still effective. It should be noticed that ${\mathbf{TBV}}_{{{\text{MIX}}}}$ aimed to reflect performance data of rust resistance in wheat, and although many rust resistance genes have been are reported for wheat (GrainGenes 2022), only two race-specific R-genes were used to simulate performance data in the study at hand, as among the plethora of R-genes for leaf, stripe, and stem rust merely few are still effective and useful for a breeding program in a given region (Flath et al. 2018; Zetzsche et al. 2019; Ghanbarnia et al. 2021). For the purpose of this study, the true breeding values ${\mathbf{TBV}}_{{{\text{MIX}}}}$ represented thus the observable genetic foreground when race-specific R-genes are still effective, while ${\mathbf{TBV}}_{{{\text{QDR}}}}$ represented the nonobservable genetic background that is masked in the presence of effective R-genes and only becomes visible if the according R-genes are overcome by the pathogen.

Assuming these race-specific R-genes are effective in the training population, the true breeding values ${\mathbf{TBV}}_{{{\text{MIX}}}}$ were subsequently used to conduct a genome-wide association mapping and fit genomic prediction models with a mixed training population ${\text{TP}}_{{{\text{MIX}}}}$ of 800 genotypes that contained both qualitative race-specific and quantitative race-nonspecific resistant lines. The genome-wide association mapping was based on a mixed linear model as implemented in the R package *sommer* (Covarrubias-Pazaran 2016), which includes a correction for kinship by the genomic relationship matrix ${\mathbf{G}}$ that was computed as suggested by (Endelman and Jannink 2012)):

3 $$G = {\text{WW}}^{{\text{T}}} /2\Sigma \left( {1 - p_{l} } \right)p_{l}$$

where $W$ is a centered marker matrix of the *i* lines with $W_{il} = Z_{il} + 1 - 2p_{l}$ and $p_{l}$ being the allele frequency at the *l*th locus. Since no clear population structure was found in the investigated panel (Suppl. Fig. S1), adjusting for the population structure by principal components did not affect the results of genome-wide association mapping and was thus omitted in the study at hand. After applying an exploratory significance threshold of  − log10 *p *value = 3, the 21 marker–trait associations (MTA) with the highest  − log10 *p* value from each chromosome were extracted and further filtered for MTAs that explained at least 5% of the variation of ${\mathbf{TBV}}_{{{\text{MIX}}}}$ in the training population. The two most significant of the remaining marker–trait associations were used to select a reduced training population that preferably contained only quantitative resistant lines ${\text{TP}}_{{{\text{QDR}}\left( {{\text{MTA}}} \right)}}$ by entering solely lines that carried the susceptible allele at both these marker–trait associations. Alternatively, various training populations ${\text{TP}}_{{{\text{QDR}}\left( {{\text{CULL}}_{{\text{x}}} } \right)}}$, also containing preferably only quantitatively resistant genotypes, were derived by culling lines that had a true breeding value of smaller than *x* = 10%, *x* = 20%, *x* = 30%, *x* = 40%, or *x* = 50% disease severity before fitting genomic prediction models.

The different training populations ${\text{TP}}_{{{\text{MIX}}}}$, ${\text{TP}}_{{{\text{QDR}}\left( {{\text{MTA}}} \right)}}$, and ${\text{TP}}_{{{\text{QDR}}\left( {{\text{CULL}}_{{\text{x}}} } \right)}}$ were subsequently used to obtain genomic best linear unbiased predictions (GBLUP) with a linear mixed model of the form:

4 $$y = Xb + Zg + e$$

where $y$ is the vector of true breeding values ${\mathbf{TBV}}_{{{\text{MIX}}}}$ and $X$ the fixed effect matrix with the corresponding vector $b$ that models the grand mean. $Z$ designated the random effect design matrix with $a$ being the vector of additive genetic line effects following $g{ }\sim { }N\left( {0,{ }G\sigma_{G}^{2} } \right)$.

For the purpose of integrating the above-mentioned two most significant marker–trait associations into the genomic predictions they were modeled as additional fixed effects when fitting models with the mixed training population ${\text{TP}}_{{{\text{MIX}}}}$:

5 $$y = Xb + M_{{{\text{TP}}}} a + Zg + e$$

where $a$ is the vector of marker effects and $M_{{{\mathbf{TP}}}}$ its corresponding design matrix for the training population, while all other design effects retain their previous designations. The genomic estimated breeding values for each genotype from this weighted genomic best linear unbiased predictions (WBLUP) model were accordingly computed as:

6 $${\text{GEBV}}_{i} = \mu + g_{i} + \mathop \sum \limits_{j = 1}^{n} m_{ij} \beta_{j}$$

where $\mu$ is the grand mean, $g_{i}$ the additive genetic effect of the *i*th genotype, $\beta_{j}$ the effect of the *j*th marker modeled as fixed effect, and $m_{ij}$ the corresponding allele call at the *j*th marker for the *i*th genotype in the validation population. Since the effects of these markers associated with the simulated R-genes were not shrunken toward zero they can be considered as being *upweighted* in the computation of the genomic estimated breeding values (Bernardo 2014; Zhao et al. 2014). Modified weighted genomic best linear unbiased predictions (mWBLUP) were furthermore computed using the same model [4] and mixed training population ${\text{TP}}_{{{\text{MIX}}}}$:

7 $${\text{GEBV}}_{i} = \mu + g_{i}$$

where $\mu$ is the grand mean and $g_{i}$ the additive genetic effect of the *i*th genotype in the validation population, but excluding and thus *unweighting* (or adjusting for) the effect of the markers associated with the simulated R-genes when computing genomic estimated breeding values. This method was considered as a computational alternative to the usage of the training populations ${\text{TP}}_{{{\text{QDR}}\left( {{\text{MTA}}} \right)}}$ and ${\text{TP}}_{{{\text{QDR}}\left( {{\text{CULL}}_{{\text{x}}} } \right)}}$, which were designed to be preferably devoid of qualitative race-specific R-genes.

It should be noticed that true breeding values, which have a heritability of $h^{2} = 1$, were used as a simplification for genome-wide association mapping and training genomic prediction models in the study at hand, since the interest was mostly in assessing the maximal potential of genomic prediction for combining qualitative race-specific and quantitative race-nonspecific disease resistance. Based on results from other studies (Jannink 2010; Combs and Bernardo 2013; de Beukelaer et al. 2017), it can be expected that using heritabilities of $0 < h^{2} < 1$ would result in lower prediction accuracies and a lower selectin gain but not in a change of ranking between the tested methods. Genomic estimated breeding values of lines in the validation population ${\mathbf{GEBV}}_{{{\text{VP}}}}$ were firstly validated by correlating them with the true breeding values ${\mathbf{TBV}}_{{{\text{MIX}}_{{{\text{VP}}}} }}$ that reflected the genetic foreground of the validation population, in a scenario where the same race-specific R-genes are effective in both the training and validation population $r_{{{\text{MIX}}}} = r\left( {{\mathbf{GEBV}}_{{{\text{VP}}}} ;{\mathbf{TBV}}_{{{\text{MIX}}_{{{\text{VP}}}} }} } \right)$. The possibility to predict the quantitative disease resistance in the genetic background of the validation population was accordingly tested by assessing the correlation $r_{{{\text{QDR}}}} = r\left( {{\mathbf{GEBV}}_{{{\text{VP}}}} ;{\mathbf{TBV}}_{{{\text{QDR}}_{{{\text{VP}}}} }} } \right)$, where ${\mathbf{TBV}}_{{{\text{QDR}}_{{{\text{VP}}}} }}$ are the true breeding values of the race-nonspecific quantitative disease resistance of the validation population. All models for the purpose of genomic prediction were fitted with the *R* package *sommer* (Covarrubias-Pazaran 2016).

### Recurrent genomic selection

**Fig. 2:**
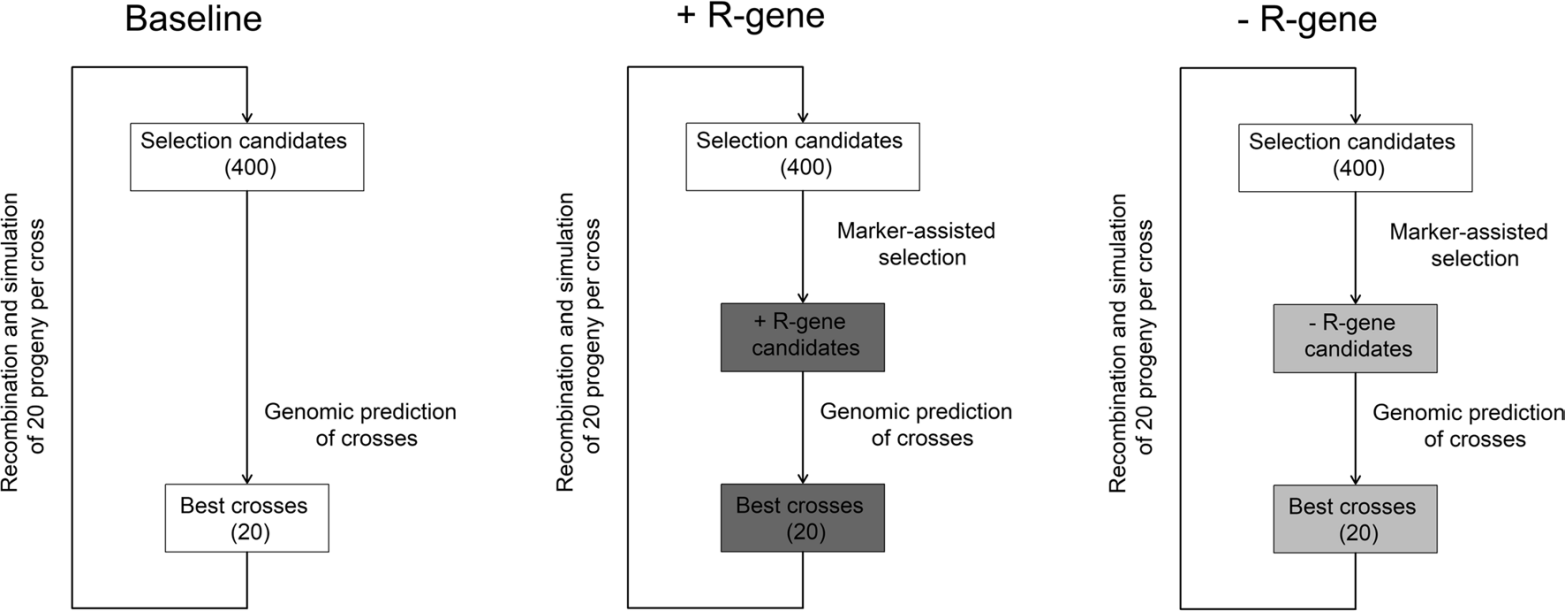
Representation of the simulated recurrent selection scheme employing either a baseline strategy using the different model-by-training populations (see also Fig. 1) for a genomic prediction of the best crosses by the mid-parent value among all possible combinations in the entire set of parents or a marker-assisted preselection among the potential parents for the presence of race-specific resistant lines (+ R-gene strategy) or the absence of race-specific resistant lines (−R-gene strategy) before genomically predicting the cross performance by the mid-parent value. It was assumed that the race-specific R-genes are still effective in the entire training population when fitting genomic prediction and identifying marker–trait associations for the preselection of parents, while the selection gain was investigated both when race-specific R-genes were considered to be effective in progeny populations (genetic foreground) and when these race-specific R-genes would have been overcome by the pathogen in the progeny populations (genetic background)

The above-described genomic prediction models and training populations were subsequently tested in a simulated recurrent selection scheme (Fig. 2), where the above-described validation populations of 400 lines served as founder populations in each of the 50 repetitions. Genomic estimated breeding values were firstly computed for these lines using the different training populations TP_MIX_, ${\text{TP}}_{{{\text{QDR}}\left( {{\text{MTA}}} \right)}}$, or ${\text{TP}}_{{{\text{QDR}}\left( {{\text{CULL}}_{{\text{x}}} } \right)}}$ and genomic prediction models described above. The best 20 crosses among these 400 potential parents were subsequently determined by estimating the genomic mid-parent value of all possible cross combinations, and 20 completely homozygous progenies were simulated for each of these crosses using a Stahl model (Stahl 1979) as implemented in the *R*/qtl package (Broman et al. 2003). The best 20 crosses between the 400 lines in this progeny population were again determined by the genomic estimated mid-parent values based on the different model-by-training population combinations to start the next selection cycle and simulate again 20 completely homozygous progenies per cross. This scheme was repeated until ten selection cycles were completed in order to investigate the long-term dynamics of genomic selection with each of the model-by-training population combinations.

This baseline genomic selection strategy was compared with two additional scenarios where the founder and progeny populations were preselected based on the marker–trait associations identified by genome-wide association mapping in the entire training population of 800 lines. The first scenario featured a preselection for lines carrying the favorable allele at these markers in either the homozygous or heterozygous state for both marker–trait associations, aiming to rapidly fix these alleles while assessing the potential of the different prediction model-by-training population combinations for improving the partial quantitative disease resistance that might also be present in the background of qualitative race-specific resistant lines (+R-gene strategy). It should be noticed that the aim in this +R-gene strategy was the preselection of parents that combined both R-genes, since it is generally recommended in practical applications, for example when breeding for rust resistance in wheat (Evanega et al. 2014), to pyramid the favorable alleles at multiple resistance genes in order to delay the evolution of virulent pathogen races. The second scenario featured in contrast a preselection for lines that carried the unfavorable allele at the detected marker–trait associations in order to assess the potential of intentionally eliminating race-specific R-gene-mediated resistance from the founder and progeny populations, and solely rely on improving the more durable but incomplete quantitative disease resistance (−R-gene strategy). It was assumed that the race-specific R-genes are effective in the entire training population of 800 lines when fitting the genomic prediction models, while the average disease resistance in each selection cycle was investigated for the case when race-specific R-genes would still provide a complete protection against an infection with the pathogen (genetic foreground) as well as in the case when the pathogen would overcome these R-genes and only race-nonspecific resistance is providing protection against an infection with the pathogen in a quantitative manner (genetic background).

## Results

**Fig. 3:**
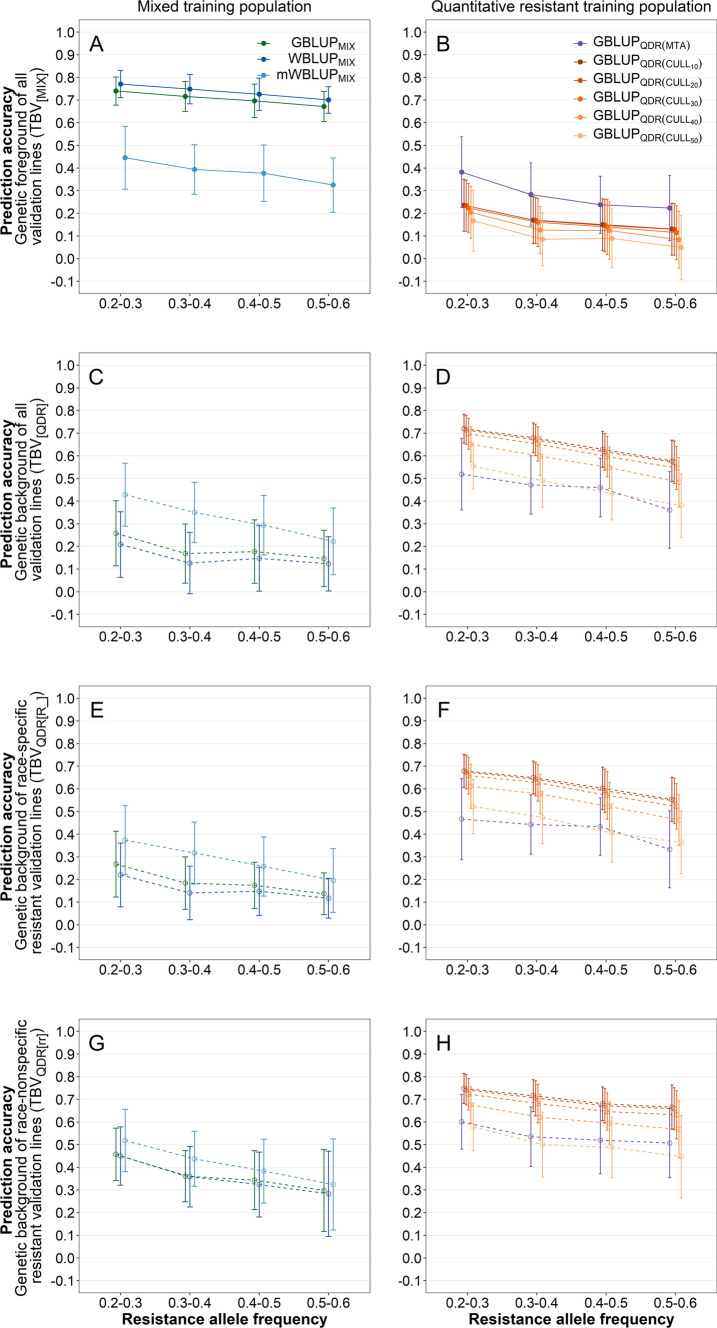
Prediction accuracy with varying allele frequencies of the simulated R-genes for the genetic foreground comprising both race-specific and race-nonspecific disease resistance, i.e., when race-specific R-genes were considered to be effective in the training populations as well as in the validation populations (${\text{TBV}}_{{{\text{MIX}}}}$) (**A**, **B**). The prediction is accuracy is furthermore shown for genetic background comprising solely the race-nonspecific quantitative disease resistance, i.e., when race-specific R-genes would not be effective anymore in the validation populations, of the entire validation population (${\text{TBV}}_{{{\text{QDR}}}}$) **C**, **D** as well as of the subpopulations of lines carrying the resistant allele **E**, **F** or the susceptible allele **G**, **H** at the simulated R-genes. It was assumed that the race-specific R-genes are still effective in the entire training population when fitting genomic best linear prediction models with a mixed training population of race-specific and race-nonspecific resistant genotypes (${\text{GBLUP}}_{{{\text{MIX}}}}$), which were compared with models including fixed effects for the most significant marker–trait associations, which were either *upweighted* (${\text{WBLUP}}_{{{\text{MIX}}}}$) or *unweighted* (${\text{mWBLUP}}_{{{\text{MIX}}}}$) in the computation of the genomic estimated breeding values. The potential of training population devoid of race-specific resistant lines was furthermore tested by removing the respective lines based on mapped marker–trait associations (${\text{GBLUP}}_{{{\text{QDR}}\left( {{\text{MTA}}} \right)}}$) or with a disease severity smaller than *x* = 10%, *x* = 20%, *x* = 30%, *x* = 40%, or *x* = 50% (${\text{GBLUP}}_{{{\text{QDR}}\left( {{\text{CULL}}_{{\text{x}}} } \right)}}$)

The simulations showed a high prediction accuracy of the GBLUP model with a mixed training population of race-specific qualitative and race-nonspecific quantitative resistant genotypes (GBLUP_MIX_) if the R-genes were effective in both the training and validation population, i.e., the aim was predicting the genetic foreground of the validation population (Fig. 3A). *Upweighting* the mapped marker–trait association (WBLUP_MIX_) led furthermore to an increase of the prediction accuracy for the genetic foreground of the validation population. Regarding these loci as noise and aiming to predict the quantitative disease resistance in the background by *unweighting* the mapped marker–trait association in the computation of genomic breeding values (mWBLUP_MIX_) resulted on the other hand in a much lower prediction accuracy in this scenario (Fig. 3A). A lower prediction accuracy was likewise observed when using training populations that were devoid of qualitative disease resistance for predicting the genetic foreground of the validation population (GBLUP_QDR_) (Fig. 3B). The ranking of these model-by-training population combinations was, however, inverted when predicting the quantitative disease resistance in the genetic background of the entire validation population (Fig. 3C, D), which reflected a scenario when race-specific R-genes are overcome by the pathogen population. The WBLUP_MIX_ model performed worst in this case (Fig. 3C), while the GBLUP_QDR_ models with different quantitatively resistant training populations showed the highest prediction accuracies for the entire validation population (Fig. 3D). The GBLUP_QDR_ model that was based on a quantitative resistant training population that contained solely lines which carried the susceptible allele at the identified marker–trait associations (${\text{GBLUP}}_{{{\text{QDR}}\left( {{\text{MTA}}} \right)}}$) showed thereby a lower prediction accuracy for the genetic background in comparison with prediction models that were based on training populations which were derived by culling lines that had a true breeding value smaller than *x* = 10%, *x *= 20%, *x* = 30%, *x* = 40%, or *x* = 50% disease severity from the original training population of 800 lines (${\text{TP}}_{{{\text{QDR}}\left( {{\text{CULL}}_{{\text{x}}} } \right)}}$) (Fig. 3D). The same observation was made for the prediction accuracy of the genetic background within the individual groups that carried either the resistant (Fig. 3E, F) or susceptible alleles (Fig. 3G, H) at the simulated qualitative resistant genes $R_{1}$ and $R_{2}$. This circumstance was also reflected by a comparably enhanced maintenance of the prediction accuracy for the quantitative (background) resistance by the GBLUP_QDR_ models based on quantitative resistant training populations in the simulated recurrent selection scheme (Suppl. Fig. S2–S4), whereas the prediction accuracy for this type of resistance diminished quickly with the GBLUP_MIX_ and WBLUP_MIX_ models based on mixed training populations of race-specific qualitative and race-nonspecific quantitative resistant genotypes.

**Fig. 4:**
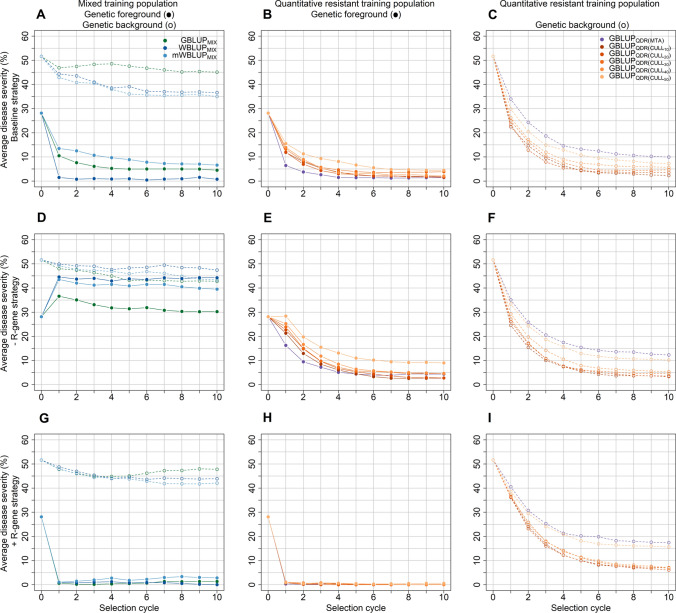
Average disease severity in the simulated recurrent selection schemes for the genetic foreground comprising both race-specific and race-nonspecific disease resistance, i.e., when race-specific R-genes were considered to be effective in the training populations as well as in the progeny populations (solid lines; closed circles) as well as the genetic background comprising solely the race-nonspecific quantitative disease resistance, i.e., when race-specific R-genes would have been overcome by the pathogen and not be effective anymore in the progeny populations (dashed lines; open circles). It was assumed that the race-specific R-genes are still effective in the entire training population when fitting genomic best linear prediction models with a mixed training population of race-specific and race-nonspecific resistant genotypes (${\text{GBLUP}}_{{{\text{MIX}}}}$), which were compared with models including fixed effects for the most significant marker–trait associations that were either *upweighted* (${\text{WBLUP}}_{{{\text{MIX}}}}$) or *unweighted* (${\text{mWBLUP}}_{{{\text{MIX}}}}$) in the computation of the genomic estimated breeding values. The potential of training population devoid of race-specific resistant lines was furthermore tested by removing the respective lines based on mapped marker–trait associations (${\text{GBLUP}}_{{{\text{QDR}}\left( {{\text{MTA}}} \right)}}$) or with a disease severity smaller than *x* = 10%, *x* = 20%, *x* = 30%, *x* = 40%, or *x* = 50% (${\text{GBLUP}}_{{{\text{QDR}}\left( {{\text{CULL}}_{{\text{x}}} } \right)}}$). The best 20 crosses among all 400 parents within each selection cycle were selected based on the genomic estimated mid-parent values obtained by the different model-by-training population combinations (**A**–**C**; baseline strategy) or after a marker-assisted preselection for the absence (**D**–**F**;−R-gene strategy) or presence (**G**–**I**; + R-gene strategy) of race-specific R-gene-mediated resistance among the potential parents. Result are shown for a resistance allele frequency of 0.2–0.3 at the simulated R-genes in the founder population

The different model-by-training population combinations were subsequently tested in a simulated recurrent selection scheme (Fig. 2), where the general aim was to identify a combination of training population design, prediction model, and selection strategy that would show the possibly highest selection gain for the disease severity in both the genetic foreground and background of the progeny populations. Hence, for each of the three tested selection strategies a comparison of training populations for the average disease severity in the genetic foreground and background seems appropriate followed by a closer look on the respective prediction models (Fig. 4).

Some selection gain was still achieved for the quantitative (background) resistance when using the GBLUP_MIX_ and WBLUP_MIX_ models with a mixed training population of qualitative and quantitative resistant lines for genomic prediction in the baseline recurrent selection scheme (Fig. 2), i.e., without any marker-assisted preselection of the parents for the absence or presence of race-specific R-genes (open circles Fig. 4A). The selection gain for the genetic background, i.e., when race-specific R-genes would not be effective anymore in the progeny populations, that was achieved by employing such a mixed training population was, however, substantially lower than the selection gain that was achieved in the genetic background by employing training populations which consisted solely of quantitative resistant lines (open circles Fig. 4A vs. open circles Fig. 4C; Suppl. Fig. S5–S7). This lower selection gain when using a mixed training population might be attributed to the discrepancy between the mixed training populations and selection candidates, since race-specific R-genes were considered to be effective in this mixed training population of qualitative and quantitative resistant lines when fitting the genomic prediction models, whereas these R-genes did not have any effect on the genetic background of the progeny population. Nevertheless, using a mixed training population of qualitative and quantitative resistant lines in combination with an *upweighting* of the mapped marker–trait association in the genomic prediction model (WBLUP_MIX_) resulted in a higher selection gain for the genetic foreground, i.e., when race-specific R-genes were considered to be effective in the training populations as well as in the progeny populations, in comparison with models trained solely with quantitative resistant lines (closed circles Fig. 4A vs. closed circles Fig. 4B).

Notwithstanding, a different pattern was observed when intentionally aiming to eliminate race-specific R-gene-mediated resistance from the progeny populations in the −R-gene strategy (Fig. 2) with a marker-assisted preselection for the absence of race-specific resistant lines among the potential parents (Fig. 4D–F). Using prediction models based on mixed training population of qualitative and quantitative resistant lines led hardly to any genetic improvement in both the genetic foreground and background when employing this strategy (closed and open circles Fig. 4D). Using training populations comprised of quantitative resistant lines resulted on the other hand in substantial genetic gain in both the genetic foreground (closed circles Fig. 4D vs. closed circles Fig. 4E) and background (open circles Fig. 4D vs. open circles Fig. 4F). However, some unexpected discrepancy between the selection gain for the foreground (${\mathbf{TBV}}_{{{\text{MIX}}}}$) and background resistance (${\mathbf{TBV}}_{{{\text{QDR}}}}$) was observed in this scenario for the model that were trained with quantitative resistant lines (Fig. 4E, F). It might be expected that aiming to eliminate race-specific R-gene resistance in the −R-gene strategy would lead to an equal selection gain in the genetic background and foreground, as the genetic background would in theory be not masked anymore by mentioned race-specific R-gene resistance. However, the R-genes were still segregating in the progeny populations when employing the −R-gene strategy, in contrast to the original intention of eliminating race-specific R-genes from the training population as well as founder populations, This issue was caused by the false classification of lines in the training population as well as founder lines of the recurrent selection scheme as being solely quantitatively resistant due to the absence of diagnostic markers for the R-genes (Suppl. Fig. S8; Suppl. File S1).

Lastly, preselecting the set of potential parents for the presence of race-specific R-genes in the + R-gene strategy (Fig. 2) and assuming they were still effective in each selection cycle resulted in a negligible disease severity in the foreground when race-specific R-genes would still be effective in the progeny population, irrespective of the model or training population that was used for predicting and selecting particular cross combinations (closed circles Fig. 4G, H). The selection gain for the quantitative resistance in the genetic background, i.e., the level of resistance that would become visible when the R-genes were to lose their effectiveness, was, however, again much higher if the prediction models were fitted with a training population that consisted of quantitatively resistant lines in comparison with a mixed training population of race-specific qualitative and race-nonspecific quantitative resistant genotypes (open circles Fig. G vs. open circles Fig. I).

## Discussion

The simultaneous presence of highly effective but potentially nondurable race-specific qualitative resistance and the more durable but incomplete race-nonspecific quantitative disease resistance (QDR) renders the choice of the most efficient resistance breeding strategy for pathosystems with strong race dynamics an inherently difficult task. One reason is given by the complex genetic architecture race-specific R-genes that provide a complete protection against an infection with the pathogen are co-occurring with many minor to medium effect loci are influencing the disease resistance in a quantitative manner, which leads to a typically right skewed distribution of performance values as often seen in empirical studies for rust resistance in wheat (Zetzsche et al. 2019; Beukert et al. 2020a; Shahinnia et al. 2022). Although these individual R-genes can already explain a large part of the genetic variation, regarding them in combination with the many race-nonspecific quantitative disease resistance loci can increase the explained genetic variation substantially, for example when considering approximately hundred stripe rust resistance loci simultaneously that were distributed all over the wheat genome (Maccaferri et al. 2015; Bulli et al. 2016) as assumed in the study at hand. An especially useful tool to support selection decisions for traits with such inheritance patterns is given by genomic prediction, due to its ability to target both large effect and many small and medium effect loci underlying complex trait genetic architectures (Ornella et al. 2012; Rutkoski et al. 2015a; Juliana et al. 2017).

The specific outcome of this endeavor, with regard to the selection for QDR and race-specific R-gene-mediated resistance appeared, however, to be highly dependent on the employed training population and prediction model, when following a genomic selection strategy in which selection decisions are solely based on genomic estimated breeding values. It was evident that following the predictions made by a GBLUP_MIX_ model, which was fitted with a mixed training population of quantitative as well as race-specific resistant lines, generally favored the selection of genotypes that carried the resistant alleles at the simulated race-specific R-genes $R_{1}$ and $R_{2}$ (Suppl. Fig. S9) irrespective of the quantitative disease resistance in their genetic background (Suppl. Fig. S10). Employing such a mixed training population drove the resistant alleles at these R-genes furthermore rapidly toward fixation after completing a few selection cycles in the simulated recurrent genomic selection, especially when upweighting putatively linked markers found by genome-wide association mapping as fixed effects in a WBLUP_MIX_ model (Suppl. Fig. S11–S13). A high prediction accuracy and gain by genomic selection can thus be expected when the same R-genes are effective both in such a mixed training population and the population of selection candidates, as has been observed in several empirical genomic selection studies for the wheat rusts (Juliana et al. 2017; Azizinia et al. 2020). Since marker-assisted or genomic predictions based on a mixed training population with different types of resistance emphasizes the selection of race-specific resistant genotypes, such a strategy can be considered a suitable short-term option in the light of the above-mentioned race dynamics of pathogen populations and the occurrence of new virulent pathogen races that are able to overcome race-specific R-genes. However, hardly any genetic improvement was observed for the QDR in the genetic background of these race-specific resistant lines.

Focusing specifically on improving the more durable QDR represents on the other hand rather a long-term strategy, and it has even been suggested to intentionally eliminate R-genes from a program´s gene pool and solely breed for QDR (Turkensteen 1993; Landeo et al. 1995). Notably, genomic selection experiments with such populations that were devoid of specific R-genes gave promising results with regard to the improvement of QDR against the obligate biotroph stem rust fungus in wheat (Rutkoski et al. 2011, 2015b). Similar observations were made when intentionally eliminating race-specific R-gene-mediated resistance from the founder and progeny populations by a marker-assisted preselection in the simulated recurrent genomic selection (−R-gene strategy) (Fig. 4D–F). The outcome when following this strategy was though again highly dependent on the employed training population. Fitting prediction models with training population that were designed to contain only QDR lines were able to facilitate a substantial selection gain in the QDR progeny populations across several selection cycles. The inclusion of race-specific resistant lines into the training population appeared on the other hand to be detrimental when following this approach, as hardly any genetic improvement was achieved when employing mixed training populations of quantitative as well as race-specific resistant lines in this or the above-described genomic selection strategy. These results suggest that an adequate training population design is pivotal in order to obtain a strong response to genomic selection for QDR, while race-specific resistant genotypes should ideally be absent from the training population for this purpose. One possibility for reaching this goal was given by conducting a marker-assisted preselection based on SNP markers that were linked with the simulated R-genes for removing race-specific genotypes from the training population.

It should, however, be noticed that in the conducted simulations the trait genetic architecture was known and genome-wide association mapping was able to detect markers that were linked to the simulated R-genes in the majority of cases (Suppl. Fig. S14; Suppl. File S1), which made the identification of race-specific resistance genotypes a straightforward task. Assuming they are still informative for the trait of interest, markers that are associated with well-defined R-genes might be used to guide an appropriate training population design in practical applications. Nevertheless, the incomplete linkage between the detected markers and the causal genes rendered the complete removal of R-gene-mediated resistance from both the training population and selection population a difficult task when purely relying on genomic information (Suppl. Fig. S12). An alternative is given by firstly defining a cutoff value based on phenotypic evaluations, which separates R-gene resistant genotypes from purely quantitative resistant genotypes with the latter being subsequently considered as selection candidates or entries of a training population. Assembling a large enough QDR training population might in this respect also pose some constrains in a genomic breeding strategy, since the training population size will decrease with an increase in the R-gene resistant allele frequency as observed in the study at hand (Suppl. Fig. S15). Additionally, QDR is for the larger part governed by many minor and medium effect loci (Stuthman et al. 2007), and accumulating them in the same genetic background in order to reach high levels of resistance would require a long-term effort. A general caveat of solely focusing on QDR and removing R-gene-mediated resistance from a breeding program can furthermore be seen in the exclusion of a substantial part of the available genetic variation for disease resistance (Stewart et al. 2003). R-genes are thus of considerable importance in breeding for disease resistance, and strategies like gene stacking and exploiting, for example epistatic interactions between multiple R-genes (Buerstmayr et al. 2014; Vazquez et al. 2015), can furthermore prolong the durability of race-specific resistance (Ellis et al. 2014) and loci underlying quantitative disease resistance like Lr34 have even been hypothesized to boost the effectiveness of R-genes in wheat (German and Kolmer 1992; Vanegas et al. 2008). Investigating the interaction between different race-specific R-genes among each other and loci underlying race-nonspecific quantitative disease resistance might thus be a rewarding topic for further genomic prediction studies focusing on resistance breeding for pathogens with strong race dynamics.

Nevertheless, integrating both above-mentioned methods of targeting R-genes and simultaneously improving the more durable QDR might represent a promising composite strategy based on the results of the study at hand (Ellis et al. 2014; Poland and Rutkoski 2016). The simulations showed accordingly that using a marker-assisted selection for R-genes in combination with a genomic prediction model based on a QDR training population represents a suitable approach to achieve this goal (+R-gene strategy) (Fig. 4G–I). The marker-assisted selection for the race-specific R-genes provided in this selection strategy a complete protection against pathogen infection in the genetic foreground of the selection candidates during the simulated recurrent genomic selection. Using genomic estimated breeding values with model-by-training population combinations that explicitly aim to improve race-nonspecific QDR led furthermore simultaneously to a strong increase of QDR in the genetic background of these lines after several cycles of selection. Applying genomic prediction models that explicitly aim to improve QDR for diseases like leaf, stem, and stripe rust in wheat in practice would moreover provide a general information about the durability of the disease resistance of pending selection candidates, and might guide selection decisions concerning the culling of the worst or selection of the best genotypes. Hence, it seems theoretically be feasible for a genomic breeding program to identify selection candidates that possess a strong QDR in their genetic background as well as a complete R-gene-mediated resistance in their genetic foreground, making the most out of disease nursery data that contains both race-specific and race-nonspecific resistance information for prediction model training. This knowledge can consequently be exploited to develop varieties and choose crossing parents, which show an increased level of resistance against pathogens with strong race dynamics and express this advantage in both the short term and long term due to a combination of a race-specific R-gene-mediated resistance with the more durable race-nonspecific QDR. Furthermore, deploying race-specific R-genes in combination with race-nonspecific QDR has the potential to delay the development of pathogen populations with multiple virulence to the employed R-genes. The suggested + R-gene strategy might in this way complement existing breeding strategies for delaying the evolution of virulent pathogen races, like stacking resistance genes as exemplified by the gene stewardship approach to reduce the genetic vulnerability of wheat to rust (Evanega et al. 2014), and provide additional information to breeders to implement and apply a knowledge-driven genomic breeding strategy for improving the resistance against diseases with strong race dynamics.

## Conclusions

Race-specific resistance genes confer complete resistance, but are often overcome when new virulent pathogen races become prevalent in a crop growing region, which leads to the classical boom-and-bust cycles. Quantitative race- nonspecific resistance (QDR) is on the other hand only partially effective, but is considered to be durable with respect to these strong race dynamics. In principle, genomic prediction offers the unique opportunity to combine both resistance types and facilitate the accumulation of the many small and medium effect loci underlying QDR to enable a long-term sustainable disease control. The theoretical framework presented in this study revealed that an appropriate training population design appeared to be pivotal for achieving this goal of being informed about the genetic background of race-specific resistant selection candidates in order to guide selection decisions in a genomic breeding program. The practical application of such a knowledge-driven genomic breeding strategy offers the opportunity to develop varieties with multiple layers of resistance, which have the potential to prevent catastrophic crop losses by delaying the development of virulent pathogen populations and by displaying a high level of QDR in the case that race-specific R-genes are overcome by evolving pathogen populations, but has to be further validated in empirical experiments.

## Supplementary Information

Below is the link to the electronic supplementary material.

Supplementary file1 (XLSX 61 KB)

Supplementary file2 (PDF 17472 KB)

## Data Availability

The datasets generated during and/or analyzed during the current study are available from the corresponding author on reasonable request.

## References

[CR1] Azizinia S, Bariana H, Kolmer J (2020). Genomic prediction of rust resistance in tetraploid wheat under field and controlled environment conditions. Agronomy.

[CR2] Bernardo R (2014). Genomewide selection when major genes are known. Crop Sci.

[CR3] Beukert U, Liu G, Thorwarth P (2020). The potential of hybrid breeding to enhance leaf rust and stripe rust resistance in wheat. Theor Appl Genet.

[CR4] Beukert U, Thorwarth P, Zhao Y (2020). Comparing the potential of marker-assisted selection and genomic prediction for improving rust resistance in hybrid wheat. Front Plant Sci.

[CR5] Broman KW, Wu H, Sen S, Churchill GA (2003). R/qtl: QTL mapping in experimental crosses. Bioinform.

[CR6] Buerstmayr M, Matiasch L, Mascher F (2014). Mapping of quantitative adult plant field resistance to leaf rust and stripe rust in two European winter wheat populations reveals co-location of three QTL conferring resistance to both rust pathogens. Theor Appl Genet.

[CR7] Bulli P, Zhang J, Chao S, Chen X (2016). Genetic architecture of resistance to stripe rust in a global winter wheat germplasm collection. G3 Genes Genomes Genet.

[CR8] Combs E, Bernardo R (2013). Accuracy of genomewide selection for different traits with constant population size, heritability, and number of markers. Plant Genome.

[CR9] Covarrubias-Pazaran G (2016). Genome-assisted prediction of quantitative traits using the R package sommer. PLoS ONE.

[CR10] Cowger C, Brown JKM (2019). Durability of quantitative resistance in crops: greater than we know?. Annu Rev Phytopathol.

[CR11] de Beukelaer H, Badke Y, Fack V, de Meyer G (2017). Moving beyond managing realized genomic relationship in long-term genomic selection. Genetics.

[CR12] Diversity arrays technology Pty Ltd (2020) DArT P/L

[CR13] Diversity arrays technology Pty Ltd (2021) A consensus map of wheat V 4.0

[CR14] Ellis JG, Lagudah ES, Spielmeyer W, Dodds PN (2014). The past, present and future of breeding rust resistant wheat. Front Plant Sci.

[CR15] Endelman JB, Jannink J-L (2012). Shrinkage estimation of the realized relationship matrix. G3 Genes Genomes Genet.

[CR16] Evanega SD, Singh RP, Coffman R, Pumphrey MO (2014). The borlaug global rust initiative: reducing the genetic vulnerability of wheat to rust. Genomics of plant genetic resources.

[CR17] Flath K, Miedaner T, Olivera PD (2018). Genes for wheat stem rust resistance postulated in German cultivars and their efficacy in seedling and adult-plant field tests. Plant Breed.

[CR18] Flor H (1955). Host-parasite interactions in flax rust-its genetics and other implications. Phytopathology.

[CR19] Flor HH (1971). Current status of the gene-for-gene concept. Annu Rev Phytopathol.

[CR20] German SE, Kolmer JA (1992). Effect of gene Lr34 in the enhancement of resistance to leaf rust of wheat. Theor Appl Genet.

[CR21] Ghanbarnia K, Gourlie R, Amundsen E, Aboukhaddour R (2021). The changing virulence of stripe rust in Canada from 1984 to 2017. Phytopathology.

[CR22] GrainGenes (2022) GrainGenes database. https://wheat.pw.usda.gov/GG3/rust. Accessed 9 Jul 2022

[CR23] Herter CP, Ebmeyer E, Kollers S (2019). An experimental approach for estimating the genomic selection advantage for Fusarium head blight and Septoria tritici blotch in winter wheat. Theor Appl Genet.

[CR24] Herter CP, Ebmeyer E, Kollers S (2019). Accuracy of within- and among-family genomic prediction for Fusarium head blight and Septoria tritici blotch in winter wheat. Theor Appl Genet.

[CR25] Hovmøller MS, Walter S, Bayles RA (2016). Replacement of the European wheat yellow rust population by new races from the centre of diversity in the near-Himalayan region. Plant Pathol.

[CR26] Jannink J-L (2010). Dynamics of long-term genomic selection. Genet Sel Evol.

[CR27] Jiang Y, Schulthess AW, Rodemann B (2017). Validating the prediction accuracies of marker-assisted and genomic selection of Fusarium head blight resistance in wheat using an independent sample. Theor Appl Genet.

[CR28] Juliana P, Singh RP, Singh PK (2017). Genomic and pedigree-based prediction for leaf, stem, and stripe rust resistance in wheat. Theor Appl Genet.

[CR29] Kaur B, Bhatia D, Mavi GS (2021). Eighty years of gene-for-gene relationship and its applications in identification and utilization of R genes. J Genet.

[CR30] Laidig F, Feike T, Hadasch S (2021). Breeding progress of disease resistance and impact of disease severity under natural infections in winter wheat variety trials. Theor Appl Genet.

[CR31] Landeo J, Gastelo M, Pinedo H, Flores F, LJ D, E B, LR C (1995). Breeding for horizontal resistance to late blight in potato free of R-genes. Phytophthora infestans 150.

[CR32] Maccaferri M, Zhang J, Bulli P (2015). A genome-wide association study of resistance to stripe rust (Puccinia striiformis f. sp. tritici) in a worldwide collection of hexaploid spring wheat (Triticum aestivum L.). G3 Genes Genomes Genet.

[CR33] McDonald B (2010). How can we achieve durable disease resistance in agricultural ecosystems?. New Phytol.

[CR34] Meuwissen THE, Hayes BJ, Goddard ME (2001). Prediction of total genetic value using genome-wide dense marker maps. Genetics.

[CR35] Miedaner T, Boeven ALG, Gaikpa DS (2020). Genomics-assisted breeding for quantitative disease resistances in small-grain cereals and maize. Int J Mol Sci.

[CR36] Miedaner T, Juroszek P (2021). Climate change will influence disease resistance breeding in wheat in Northwestern Europe. Theor Appl Genet.

[CR37] Ornella L, Singh S, Perez P (2012). Genomic prediction of genetic values for resistance to wheat rusts. Plant Genome J.

[CR38] Pal N, Jan I, Saini DK (2022). Meta-QTLs for multiple disease resistance involving three rusts in common wheat (Triticum aestivum L.). Theor Appl Genet.

[CR39] Pink DAC (2002). Strategies using genes for non-durable disease resistance. Euphytica.

[CR40] Poland JA, Balint-Kurti PJ, Wisser RJ (2009). Shades of gray: the world of quantitative disease resistance. Trends Plant Sci.

[CR41] Poland J, Rutkoski J (2016). Advances and challenges in genomic selection for disease resistance. Annu Rev Phytopathol.

[CR42] Robertsen C, Hjortshøj R, Janss L (2019). Genomic Sel Cereal Breed Agron.

[CR43] Rutkoski JE, Heffner EL, Sorrells ME (2011). Genomic selection for durable stem rust resistance in wheat. Euphytica.

[CR44] Rutkoski J, Singh RP, Huerta-Espino J (2015). Efficient use of historical data for genomic selection: a case study of stem rust resistance in wheat. Plant Genome.

[CR45] Rutkoski J, Singh RP, Huerta-Espino J (2015). Genetic gain from phenotypic and genomic selection for quantitative resistance to stem rust of wheat. Plant Genome.

[CR46] Semagn K, Iqbal M, Jarquin D (2022). Genomic predictions for common bunt, FHB, stripe rust, leaf rust, and leaf spotting resistance in spring wheat. Genes (basel).

[CR47] Semagn K, Iqbal M, Jarquin D (2022). Genomic prediction accuracy of stripe rust in six spring wheat populations by modeling genotype by environment interaction. Plants.

[CR48] Shahinnia F, Geyer M, Schürmann F (2022). Genome-wide association study and genomic prediction of resistance to stripe rust in current central and Northern European winter wheat germplasm. Theor Appl Genet.

[CR49] Singh RP, Hodson DP, Jin Y (2015). Emergence and spread of new races of wheat stem rust fungus: continued threat to food security and prospects of genetic control. Phytopathology.

[CR50] Sørensen CK, Hovmøller MS, Leconte M (2014). New races of puccinia striiformis found in Europe reveal race specificity of long-term effective adult plant resistance in wheat. Phytopathology.

[CR51] Stahl FW (1979). Special sites in generalized recombination. Annu Rev Genet.

[CR52] Stewart HE, Bradshaw JE, Pande B (2003). The effect of the presence of R-genes for resistance to late blight (Phytophthora infestans) of potato (Solanum tuberosum) on the underlying level of field resistance. Plant Pathol.

[CR53] Stich B, Van Inghelandt D (2018). Prospects and potential uses of genomic prediction of key performance traits in tetraploid potato. Front Plant Sci.

[CR54] Stuthman DD, Leonard KJ, Miller-Garvin J (2007). Breeding Crops for Durable Resistance to Disease. Adv Agron.

[CR55] Turkensteen LJ, Jacobs T, Parlevliet J (1993). Durable resistance of potatoes against phytophthora infestans. Durability of disease resistance.

[CR56] van der Plank J (1963). Plant diseases: epidemics and control.

[CR57] Vanegas CDG, Garvin DF, Kolmer JA (2008). Genetics of stem rust resistance in the spring wheat cultivar Thatcher and the enhancement of stem rust resistance by Lr34. Euphytica.

[CR58] Vazquez MD, Zemetra R, Peterson CJ (2015). Multi-location wheat stripe rust QTL analysis: genetic background and epistatic interactions. Theor Appl Genet.

[CR59] Zetzsche H, Serfling A, Ordon F (2019). Breeding Progress in Seedling Resistance against Various Races of Stripe and Leaf Rust in European Bread Wheat. Crop Breed Genet Genom.

[CR60] Zhao Y, Mette MF, Gowda M (2014). Bridging the gap between marker-assisted and genomic selection of heading time and plant height in hybrid wheat. Heredity (edinb).

