# Supplementary material for: Toward combining qualitative race-specific and quantitative race-nonspecific disease resistance by genomic selection
Source: Theor Appl Genet. 2023 Mar 23;136(4):79. doi: 10.1007/s00122-023-04312-2 (PMC10036288; doi:10.1007/s00122-023-04312-2)
Supplement: Supplementary file 2 — Supplementary file2 (PDF 17472 KB) [file 122_2023_4312_MOESM2_ESM.pdf]

Supplementary material

Article Title: Towards combining qualitative race-specific and quantitative race-nonspecific disease resistance by genomic selection

Journal: Theoretical and Applied Genetics

Authors: Sebastian Michel, Franziska Löschenberger, Christian Ametz, Hermann Bürstmayr

Name, affiliation, and email of corresponding author:

Sebastian Michel
Department for Agrobiotechnology (IFA-Tulln)
Institute for Biotechnology in Plant Production
University of Natural Resources and Life Sciences, Vienna (BOKU)
Konrad-Lorenz-Str. 20, 3430 Tulln, Austria
e-mail: sebastian.michel@boku.ac.at

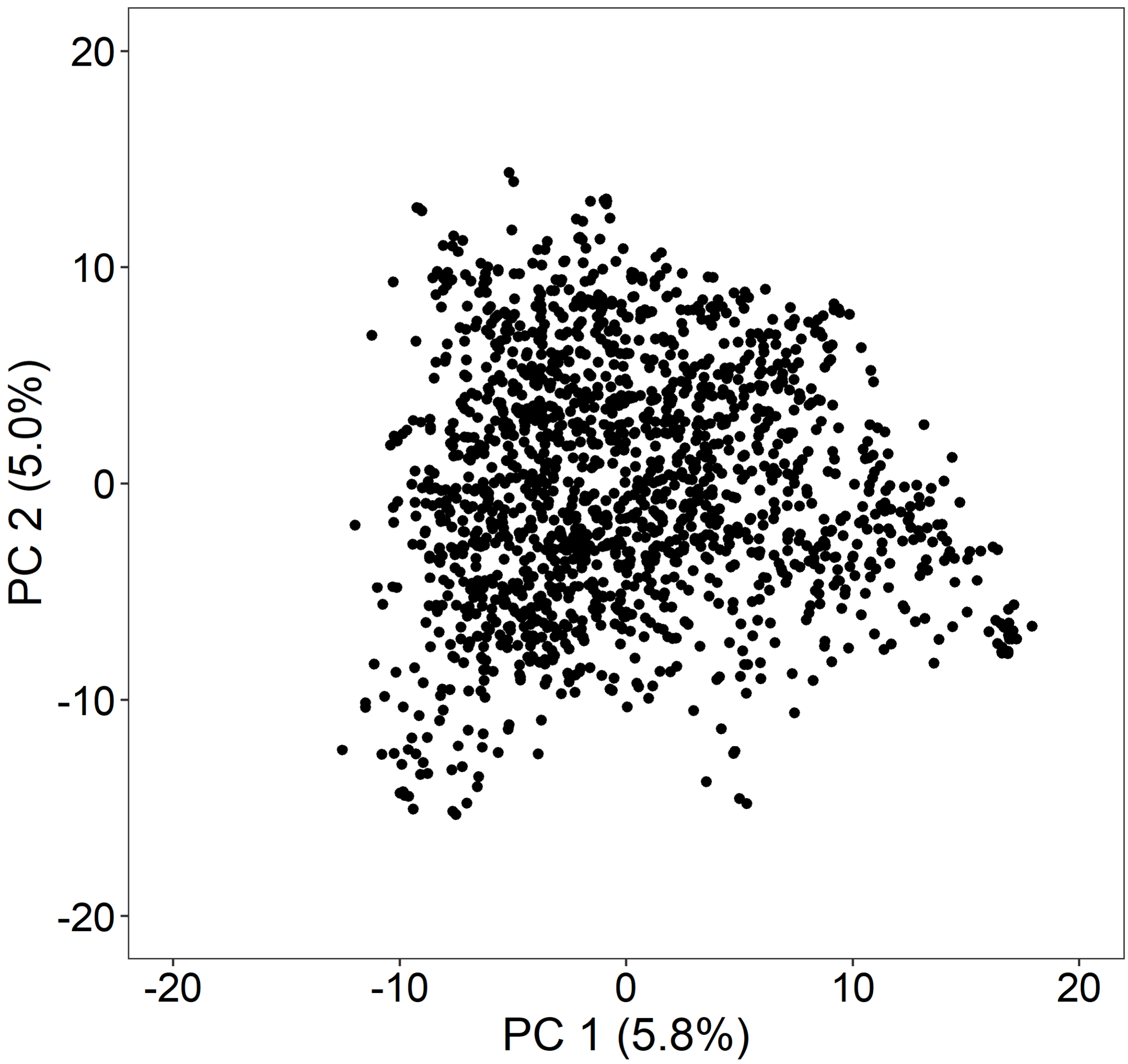

Figure S1 Principal component analysis of the 1628 lines involved in the study.

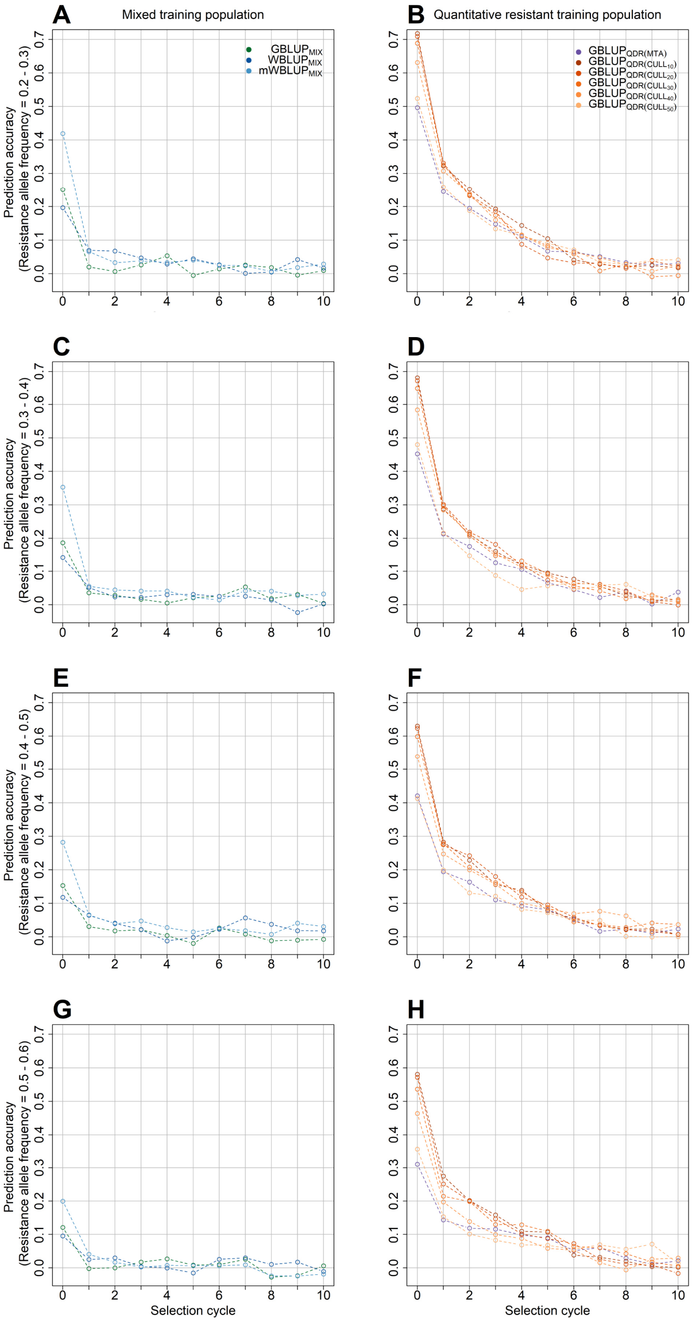

Figure S2 Average prediction accuracy in the simulated recurrent selection schemes without pre-selecting the sets of potential parents (Baseline strategy) for the genetic foreground comprising both race-specific and race-nonspecific disease resistance, i.e. when race-specific R-genes were considered to be effective in the training populations as well as in the progeny populations (solid lines; closed circles) as well as the genetic background comprising solely the race-nonspecific quantitative disease resistance, i.e. when race-specific R-genes would have been overcome by the pathogen and not be effective anymore in the progeny populations (dashed lines; open circles). It was assumed that the race-specific R-genes are still effective in the entire training population when fitting genomic best linear prediction models with a mixed training population of race-specific and race-nonspecific resistant genotypes ($\mathrm{GBLUP_{MIX}}$), which were compared with models including fixed effects for the most significant marker-trait associations that were either *upweighted* ($\mathrm{WBLUP_{MIX}}$) or *unweighted* ($\mathrm{mWBLUP_{MIX}}$) in the computation of the genomic estimated breeding values. The potential of training population devoid of race-specific resistant lines was furthermore tested by removing the respective lines based on mapped marker-trait associations ($\mathrm{GBLUP_{QDR(MTA)}}$) or with a disease severity smaller than x = 10%, x = 20%, x = 30%, x = 40%, or x = 50% ($\mathrm{GBLUP_{QDR(CULL_x)}}$). Result are shown for an initial resistance allele frequency of 0.2-0.3 (A+B), 0.3-0.4 (C+D), 0.4-0.5 (E+F), 0.5-0.6 (G+H) at the simulated R-genes in the founder population.

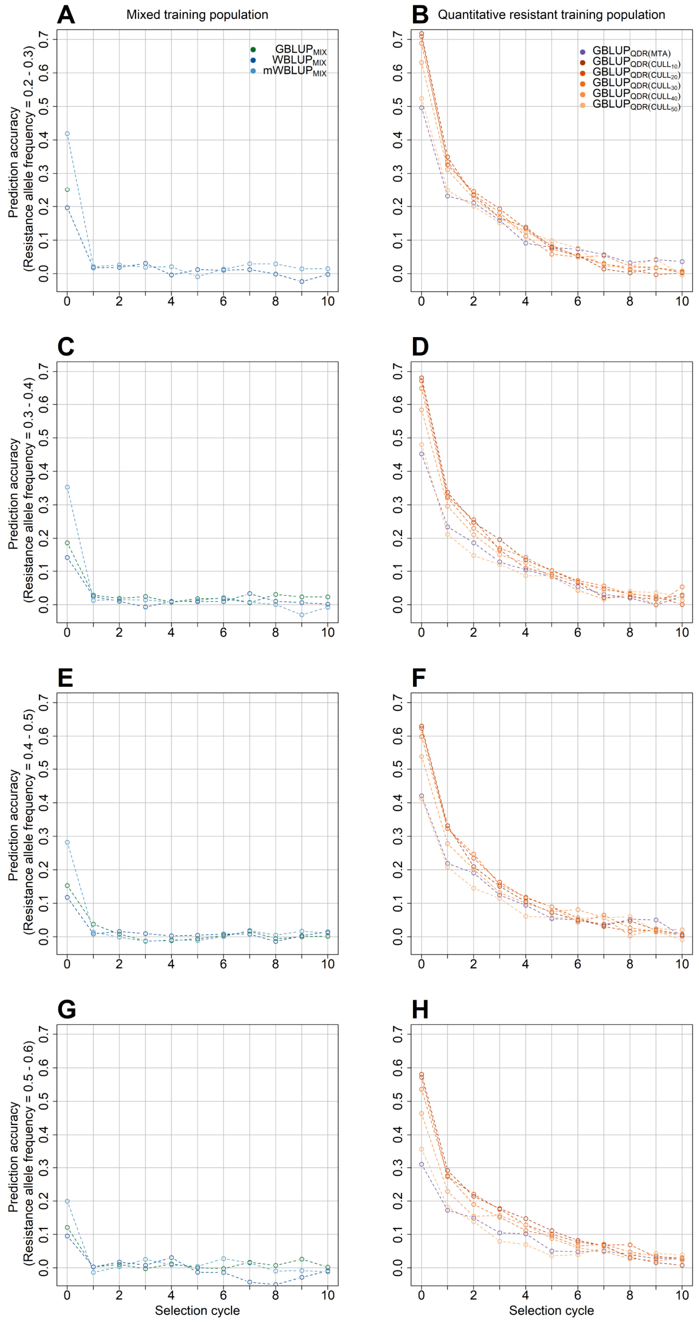

Figure S3 Average prediction accuracy in the simulated recurrent selection schemes when pre-selecting the sets of potential parents for the absence of race-specific R-gene mediated resistance (- R-gene strategy) for the genetic foreground comprising both race-specific and race-nonspecific disease resistance, i.e. when race-specific R-genes were considered to be effective in the training populations as well as in the progeny populations (solid lines; closed circles) as well as the genetic background comprising solely the race-nonspecific quantitative disease resistance, i.e. when race-specific R-genes would have been overcome by the pathogen and not be effective anymore in the progeny populations (dashed lines; open circles). It was assumed that the race-specific R-genes are still effective in the entire training population when fitting genomic best linear prediction models with a mixed training population of race-specific and race-nonspecific resistant genotypes ($\mathrm{GBLUP_{MIX}}$), which were compared with models including fixed effects for the most significant marker-trait associations that were either *upweighted* ($\mathrm{WBLUP_{MIX}}$) or *unweighted* ($\mathrm{mWBLUP_{MIX}}$) in the computation of the genomic estimated breeding values. The potential of training population devoid of race-specific resistant lines was furthermore tested by removing the respective lines based on mapped marker-trait associations ($\mathrm{GBLUP_{QDR(MTA)}}$) or with a disease severity smaller than x = 10%, x = 20%, x = 30%, x = 40%, or x = 50% ($\mathrm{GBLUP_{QDR(CULL_x)}}$). Result are shown for an initial resistance allele frequency of 0.2-0.3 (A+B), 0.3-0.4 (C+D), 0.4-0.5 (E+F), 0.5-0.6 (G+H) at the simulated R-genes in the founder population.

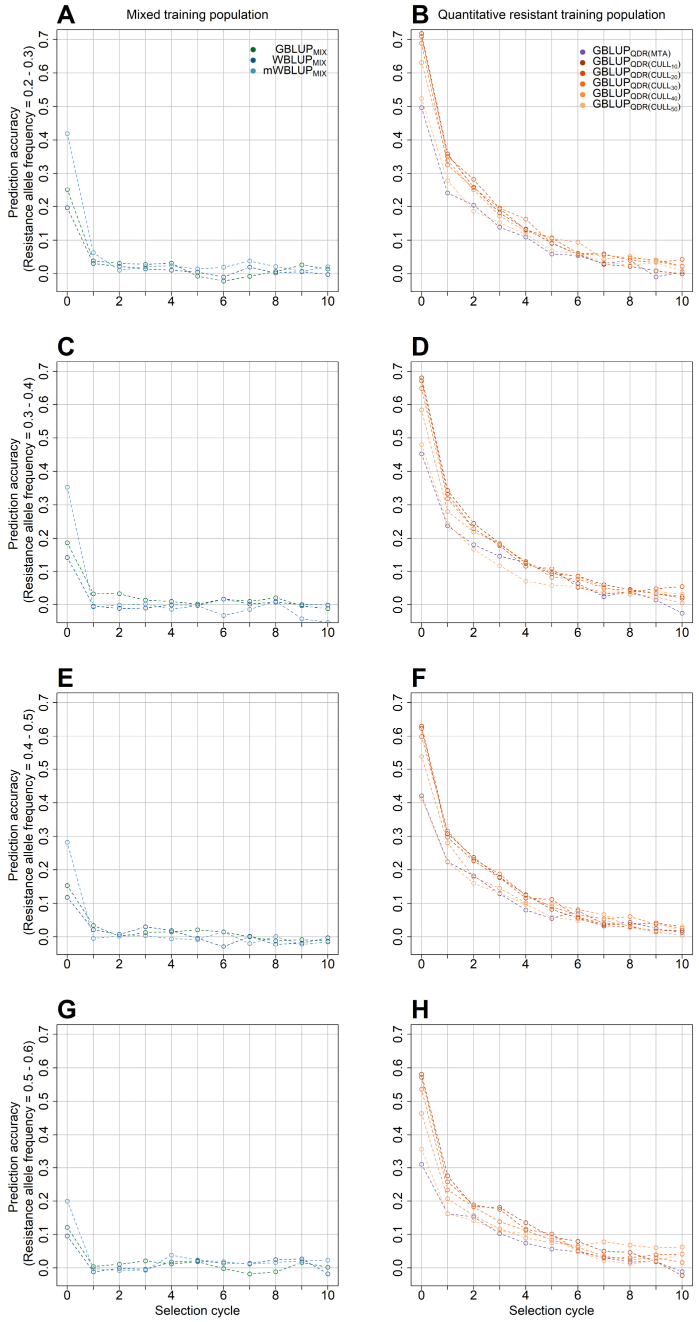

Figure S4 Average prediction accuracy in the simulated recurrent selection schemes <u>when pre-selecting the sets of potential parents for the presence of race-specific R-gene mediated resistance resistance (-R-gene strategy)</u> for the genetic foreground comprising both race-specific and race-nonspecific disease resistance, i.e. when race-specific R-genes were considered to be effective in the training populations as well as in the progeny populations (solid lines; closed circles) as well as the genetic background comprising solely the race-nonspecific quantitative disease resistance, i.e. when race-specific R-genes would have been overcome by the pathogen and not be effective anymore in the progeny populations (dashed lines; open circles). It was assumed that the race-specific R-genes are still effective in the entire training population when fitting genomic best linear prediction models with a mixed training population of race-specific and race-nonspecific resistant genotypes ($\mathrm{GBLUP_{MIX}}$), which were compared with models including fixed effects for the most significant marker-trait associations that were either *upweighted* ($\mathrm{WBLUP_{MIX}}$) or *unweighted* ($\mathrm{mWBLUP_{MIX}}$) in the computation of the genomic estimated breeding values. The potential of training population devoid of race-specific resistant lines was furthermore tested by removing the respective lines based on mapped marker-trait associations ($\mathrm{GBLUP_{QDR(MTA)}}$) or with a disease severity smaller than x = 10%, x = 20%, x = 30%, x = 40%, or x = 50% ($\mathrm{GBLUP_{QDR(CULL_x)}}$). Result are shown for an initial resistance allele frequency of 0.2-0.3 (A+B), 0.3-0.4 (C+D), 0.4-0.5 (E+F), 0.5-0.6 (G+H) at the simulated R-genes in the founder population.

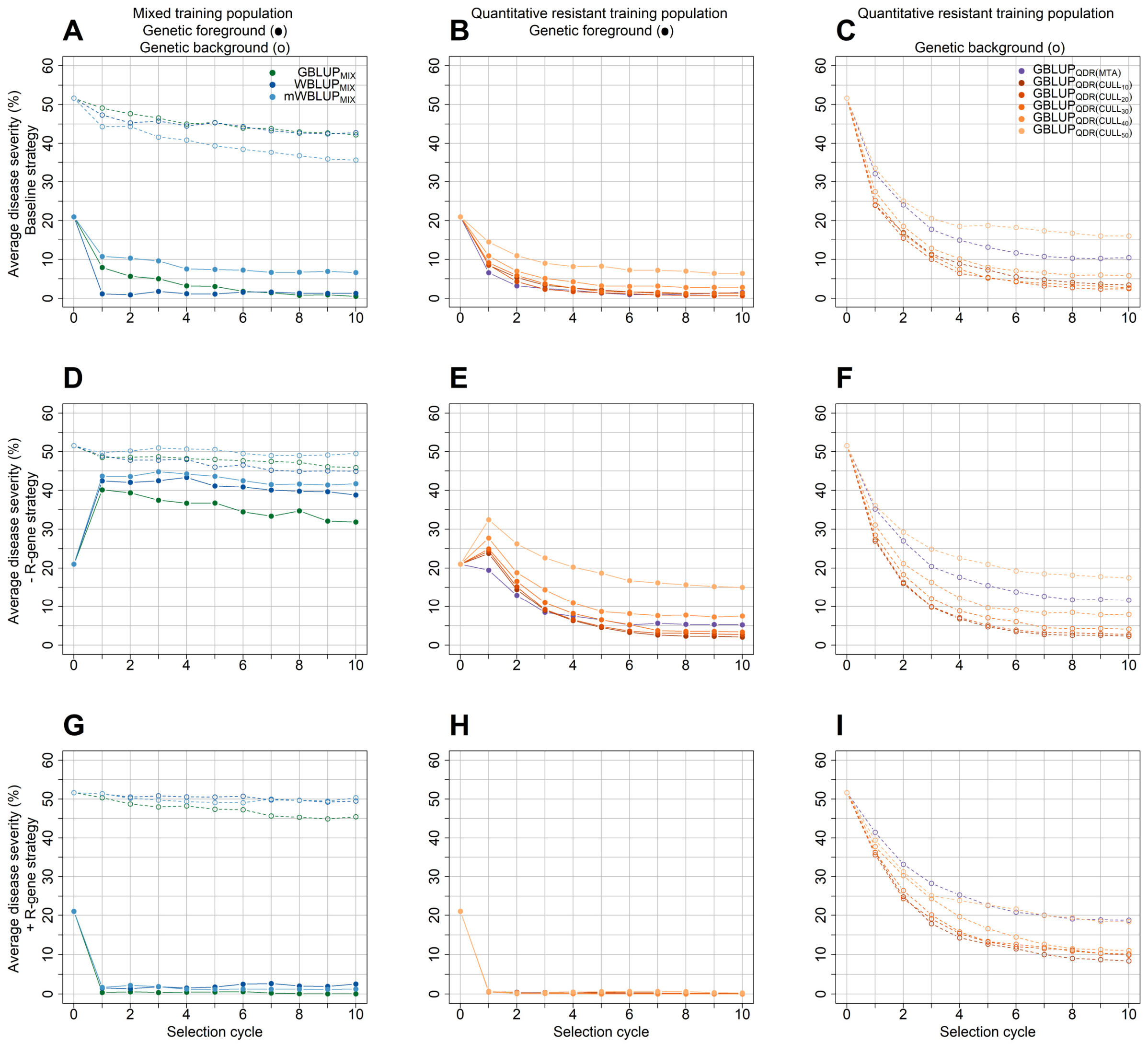

Figure S5 Average disease severity in the simulated recurrent selection schemes for the genetic foreground comprising both race-specific and race-nonspecific disease resistance, i.e. when race-specific R-genes were considered to be effective in the training populations as well as in the progeny populations (solid lines; closed circles) as well as the genetic background comprising solely the race-nonspecific quantitative disease resistance, i.e. when race-specific R-genes would have been overcome by the pathogen and not be effective anymore in the progeny populations (dashed lines; open circles). It was assumed that the race-specific R-genes are still effective in the entire training population when fitting genomic best linear prediction models with a mixed training population of race-specific and race-nonspecific resistant genotypes ($\mathrm{GBLUP_{MIX}}$), which were compared with models including fixed effects for the most significant marker-trait associations that were either *upweighted* ($\mathrm{WBLUP_{MIX}}$) or *unweighted* ($\mathrm{mWBLUP_{MIX}}$) in the computation of the genomic estimated breeding values. The potential of training population devoid of race-specific resistant lines was furthermore tested by removing the respective lines based on mapped marker-trait associations ($\mathrm{GBLUP_{QDR(MTA)}}$) or with a disease severity smaller than x = 10%, x = 20%, x = 30%, x = 40%, or x = 50% ($\mathrm{GBLUP_{QDR(CULL_x)}}$). The best 20 crosses among all 400 parents within each selection cycle were selected based on the genomic estimated mid-parent values obtained by the different model-by-training population combinations (A-C; baseline strategy) or after a marker-assisted pre-selection for the absence (D-F; - R-gene strategy) or presence (G-I; + R-gene strategy) of race-specific R-gene mediated resistance among the potential parents. Result are shown for a resistance allele frequency of 0.3-0.4 at the simulated R-genes in the founder population.

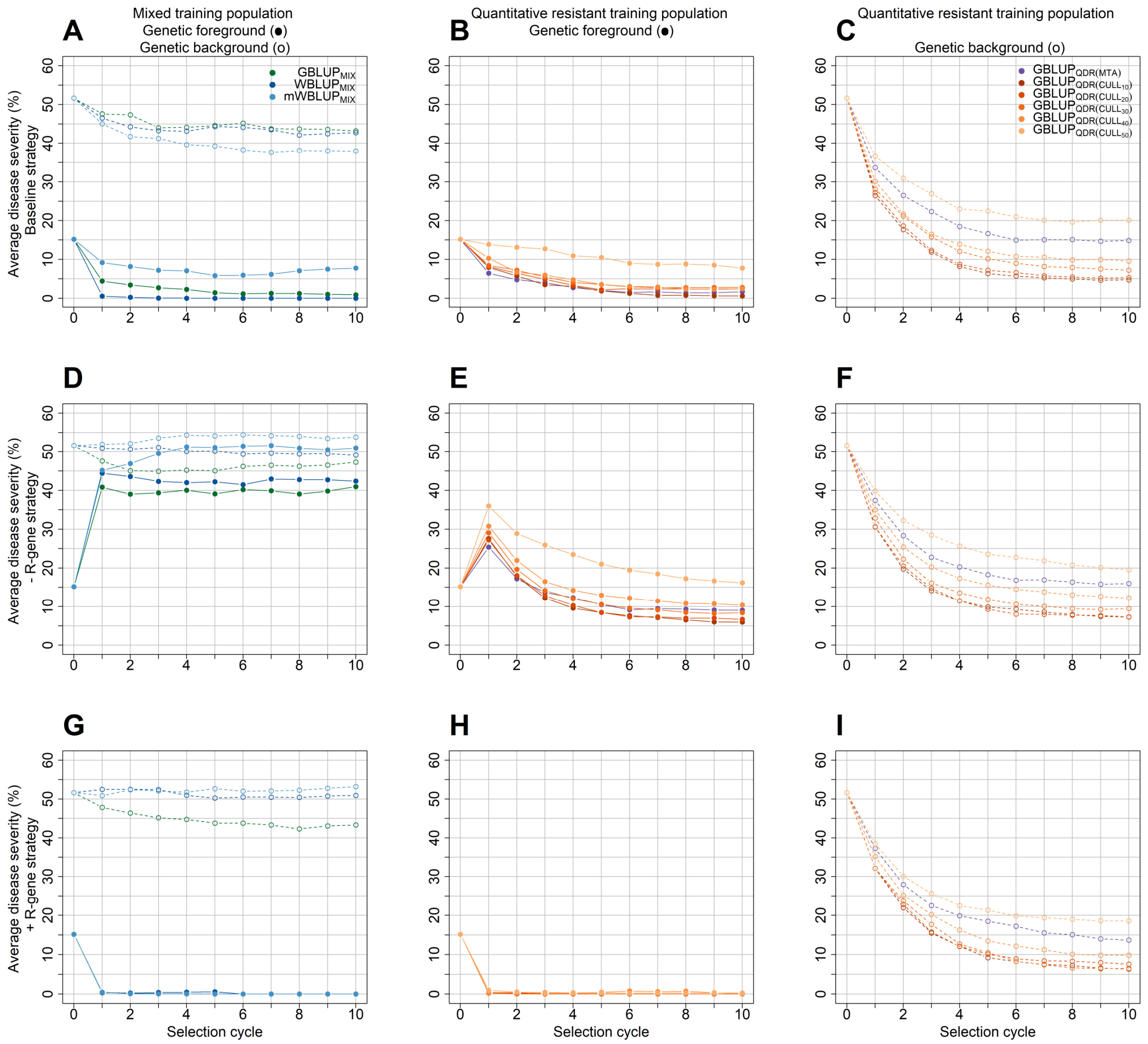

Figure S6 Average disease severity in the simulated recurrent selection schemes for the genetic foreground comprising both race-specific and race-nonspecific disease resistance, i.e. when race-specific R-genes were considered to be effective in the training populations as well as in the progeny populations (solid lines; closed circles) as well as the genetic background comprising solely the race-nonspecific quantitative disease resistance, i.e. when race-specific R-genes would have been overcome by the pathogen and not be effective anymore in the progeny populations (dashed lines; open circles). It was assumed that the race-specific R-genes are still effective in the entire training population when fitting genomic best linear prediction models with a mixed training population of race-specific and race-nonspecific resistant genotypes ($\mathrm{GBLUP_{MIX}}$), which were compared with models including fixed effects for the most significant marker-trait associations that were either *upweighted* ($\mathrm{WBLUP_{MIX}}$) or *unweighted* ($\mathrm{mWBLUP_{MIX}}$) in the computation of the genomic estimated breeding values. The potential of training population devoid of race-specific resistant lines was furthermore tested by removing the respective lines based on mapped marker-trait associations ($\mathrm{GBLUP_{QDR(MTA)}}$) or with a disease severity smaller than x = 10%, x = 20%, x = 30%, x = 40%, or x = 50% ($\mathrm{GBLUP_{QDR(CULL_x)}}$). The best 20 crosses among all 400 parents within each selection cycle were selected based on the genomic estimated mid-parent values obtained by the different model-by-training population combinations (A-C; baseline strategy) or after a marker-assisted pre-selection for the absence (D-F; - R-gene strategy) or presence (G-I; + R-gene strategy) of race-specific R-gene mediated resistance among the potential parents. Result are shown for a resistance allele frequency of 0.4-0.5 at the simulated R-genes in the founder population.

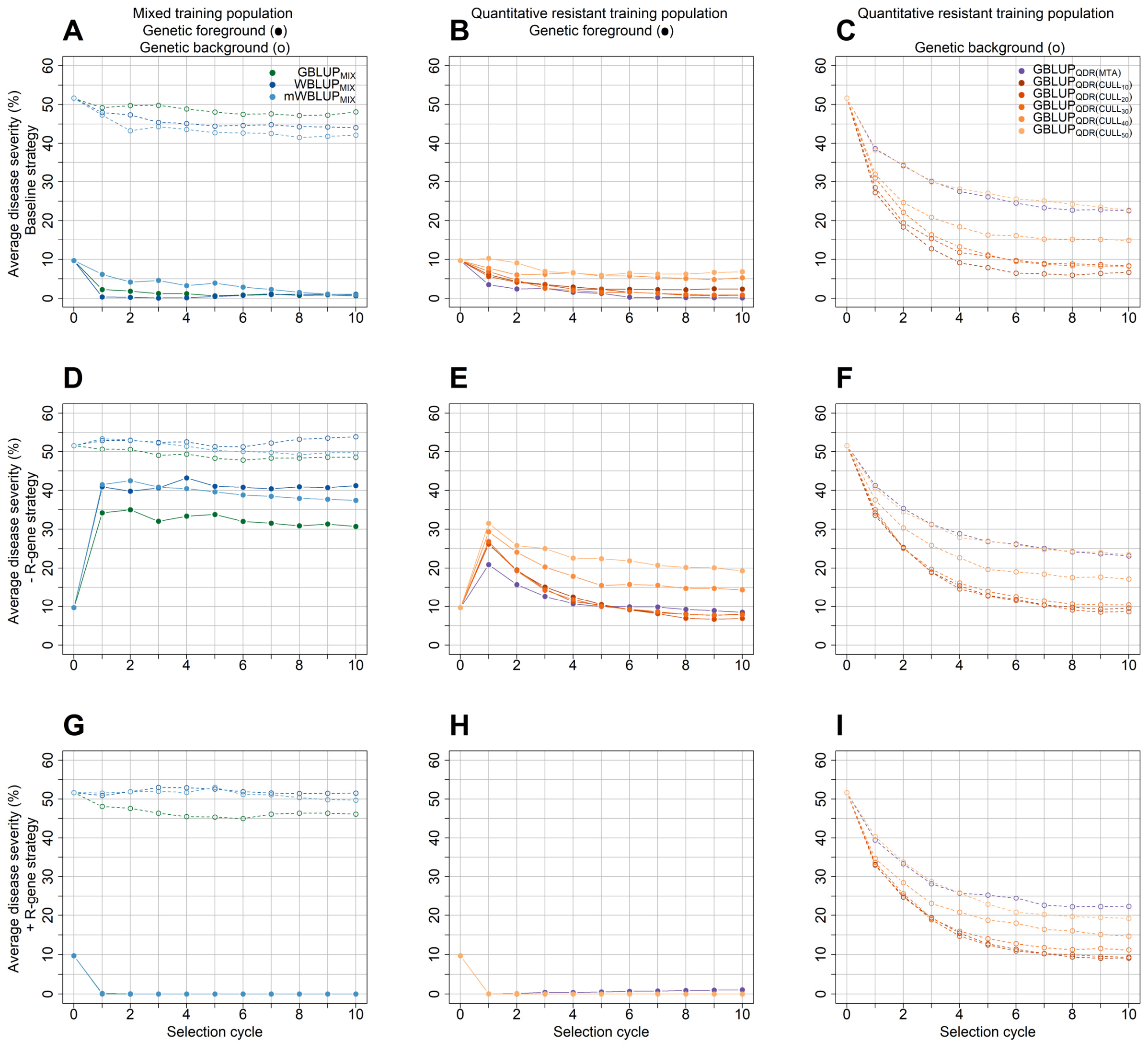

Figure S7 Average disease severity in the simulated recurrent selection schemes for the genetic foreground comprising both race-specific and race-nonspecific disease resistance, i.e. when race-specific R-genes were considered to be effective in the training populations as well as in the progeny populations (solid lines; closed circles) as well as the genetic background comprising solely the race-nonspecific quantitative disease resistance, i.e. when race-specific R-genes would have been overcome by the pathogen and not be effective anymore in the progeny populations (dashed lines; open circles). It was assumed that the race-specific R-genes are still effective in the entire training population when fitting genomic best linear prediction models with a mixed training population of race-specific and race-nonspecific resistant genotypes ($\mathrm{GBLUP_{MIX}}$), which were compared with models including fixed effects for the most significant marker-trait associations that were either *upweighted* ($\mathrm{WBLUP_{MIX}}$) or *unweighted* ($\mathrm{mWBLUP_{MIX}}$) in the computation of the genomic estimated breeding values. The potential of training population devoid of race-specific resistant lines was furthermore tested by removing the respective lines based on mapped marker-trait associations ($\mathrm{GBLUP_{QDR(MTA)}}$) or with a disease severity smaller than x = 10%, x = 20%, x = 30%, x = 40%, or x = 50% ($\mathrm{GBLUP_{QDR(CULL_x)}}$). The best 20 crosses among all 400 parents within each selection cycle were selected based on the genomic estimated mid-parent values obtained by the different model-by-training population combinations (A-C; baseline strategy) or after a marker-assisted pre-selection for the absence (D-F; - R-gene strategy) or presence (G-I; + R-gene strategy) of race-specific R-gene mediated resistance among the potential parents. Result are shown for a resistance allele frequency of 0.5-0.6 at the simulated R-genes in the founder population.

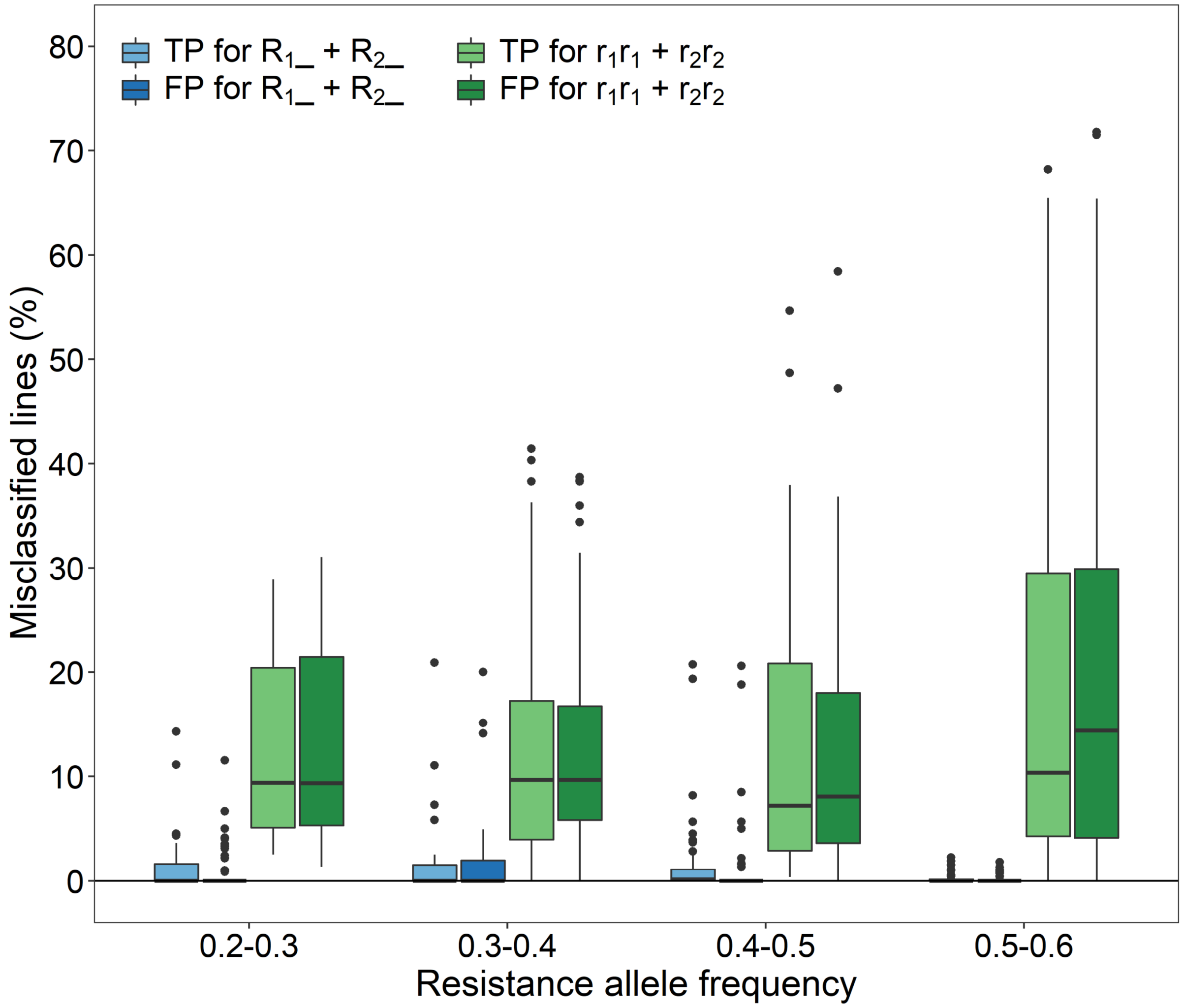

Figure S8 Percentage of misclassified lines when conducting a marker-assisted selection for the presence ($R_1\_ + R_2\_$) or absence ($r_1r_1 + r_2r_2$) of race-specific resistance in the training population (TP) and founder population (FP) of the recurrent selection scheme.

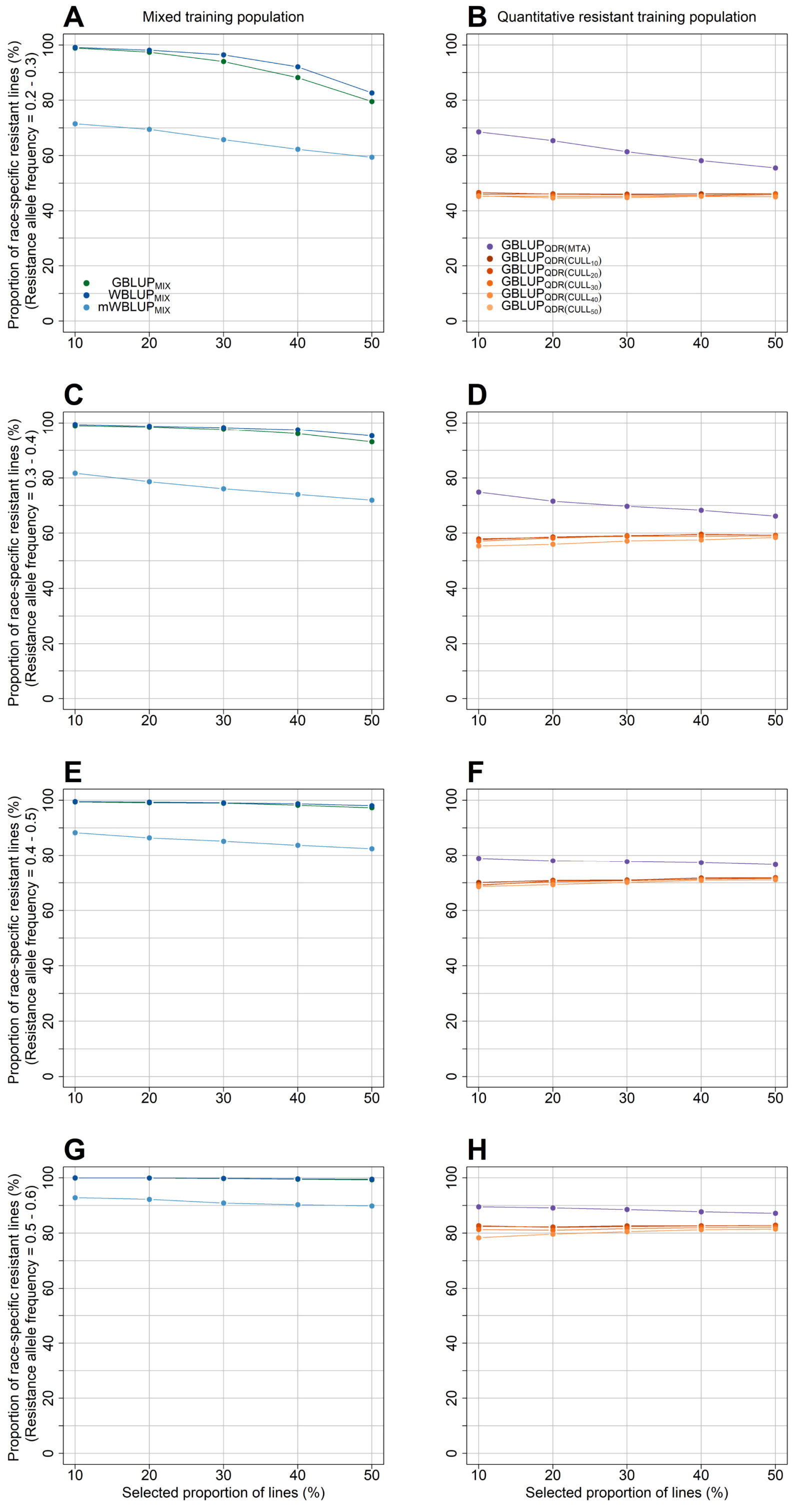

Figure S9 Averaged proportion of race-specific resistant lines among the best 10-50% genomically selected lines by the different prediction model-by-training population combinations. It was assumed that the race-specific R-genes are still effective in the entire training population when fitting genomic best linear prediction models with a mixed training population of race-specific and race-nonspecific resistant genotypes ($\mathrm{GBLUP_{MIX}}$), which were compared with models including fixed effects for the most significant marker-trait associations, which were either *upweighted* ($\mathrm{WBLUP_{MIX}}$) or *unweighted* ($\mathrm{mWBLUP_{MIX}}$) in the computation of the genomic estimated breeding values. The potential of training population devoid of race-specific resistant lines was furthermore tested by removing the respective lines based on mapped marker-trait associations ($\mathrm{GBLUP_{QDR(MTA)}}$) or with a disease severity smaller than x = 10%, x = 20%, x = 30%, x = 40%, or x = 50% ($\mathrm{GBLUP_{QDR(CULL_x)}}$). Result are shown for a resistance allele frequency of 0.2-0.3 (A+B), 0.3-0.4 (C+D), 0.4-0.5 (E+F), 0.5-0.6 (G+H) at the simulated R-genes in the validation population.

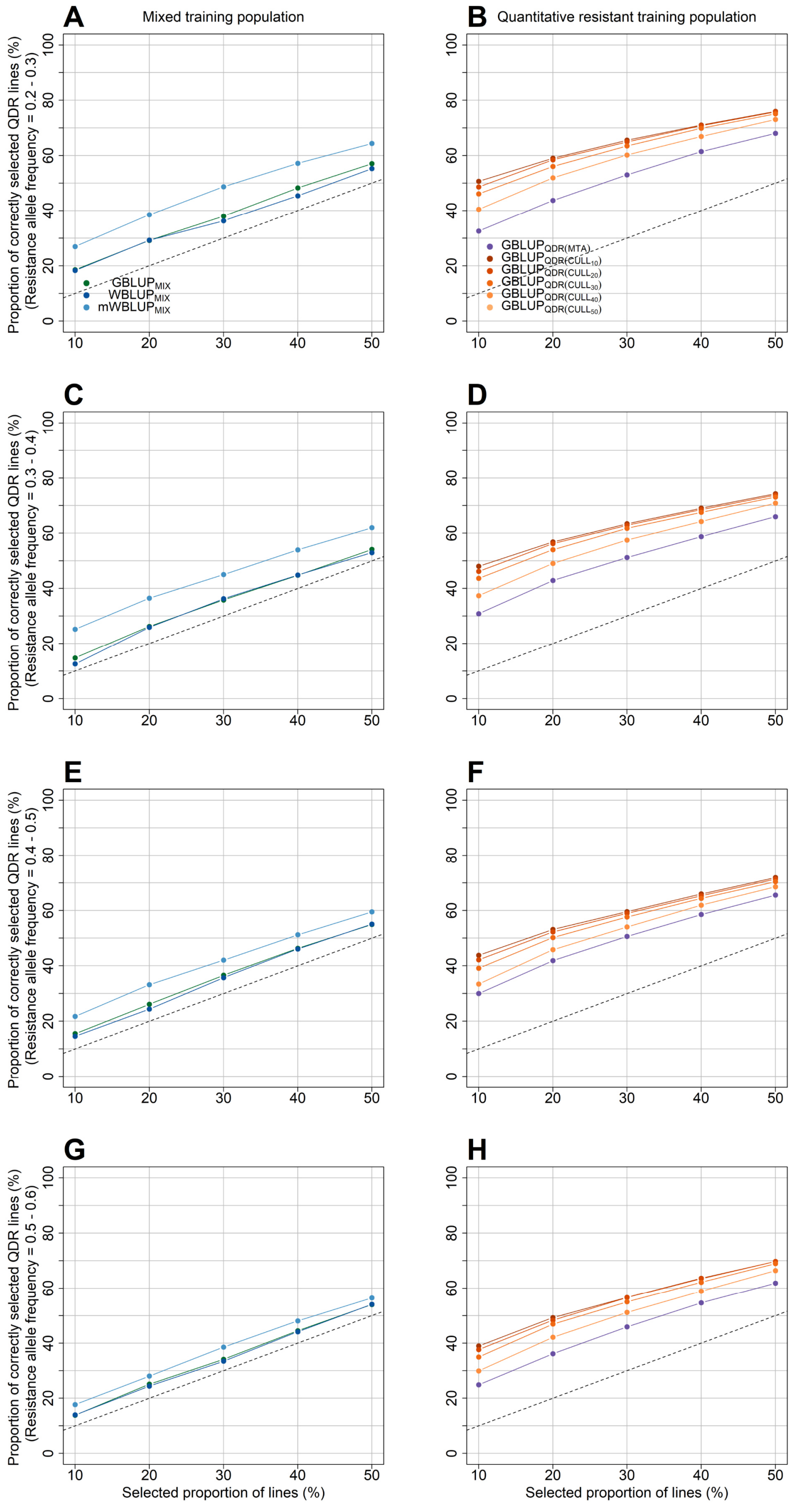

Figure S10 Averaged percentage of correctly selected best race-nonspecific quantitative resistant (QDR) lines among the best 10-50% genomically selected lines by the different prediction model-by-training population combinations. It was assumed that the race-specific R-genes are still effective in the entire training population when fitting genomic best linear prediction models with a mixed training population of race-specific and race-nonspecific resistant genotypes ($\mathrm{GBLUP_{MIX}}$), which were compared with models including fixed effects for the most significant marker-trait associations, which were either *upweighted* ($\mathrm{WBLUP_{MIX}}$) or *unweighted* ($\mathrm{mWBLUP_{MIX}}$) in the computation of the genomic estimated breeding values. The potential of training population devoid of race-specific resistant lines was furthermore tested by removing the respective lines based on mapped marker-trait associations ($\mathrm{GBLUP_{QDR(MTA)}}$) or with a disease severity smaller than x = 10%, x = 20%, x = 30%, x = 40%, or x = 50% ($\mathrm{GBLUP_{QDR(CULL_x)}}$). Result are shown for a resistance allele frequency of 0.2-0.3 (A+B), 0.3-0.4 (C+D), 0.4-0.5 (E+F), 0.5-0.6 (G+H) at the simulated R-genes in the validation population.

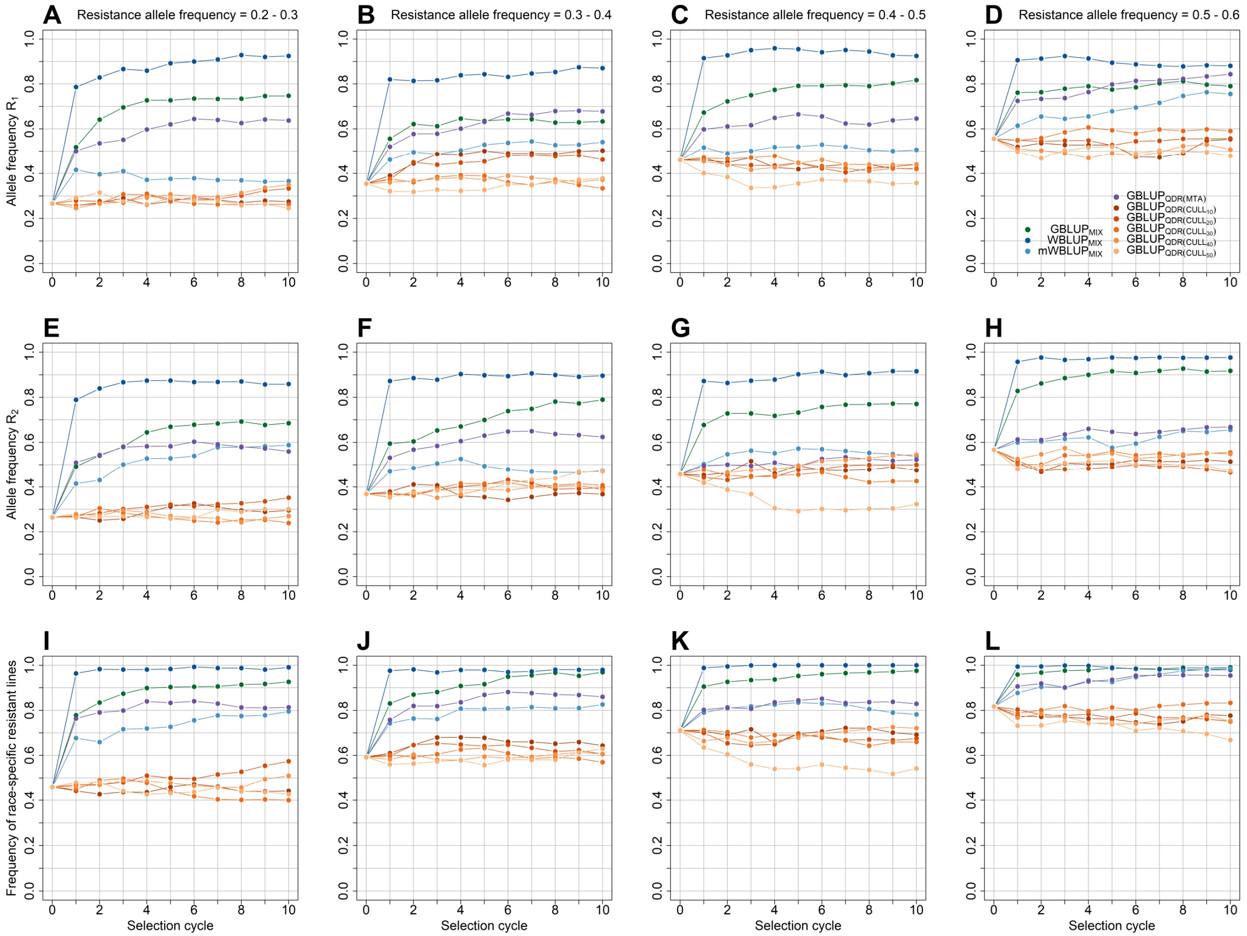

Figure S11 Frequency of the resistant allele R1 (A-D) and R2 (E-H) at the simulated race-specific R-genes as well as the frequency of race-specific resistant lines (**I**-L) in the recurrent selection without pre-selecting the sets of potential parents (Baseline strategy). Result are shown for a resistance allele frequency of 0.2-0.3 (A+E+**I**), 0.3-0.4 (B+F+J), 0.4-0.5 (C+G+K), and 0.5-0.6 (D+H+L) at the simulated R-genes in the founder population. It was assumed that the race-specific R-genes are still effective in the entire training population when fitting genomic best linear prediction models with a mixed training population of race-specific and race-nonspecific resistant genotypes ($\mathrm{GBLUP_{MIX}}$), which were compared with models including fixed effects for the most significant marker-trait associations, which were either *upweighted* ($\mathrm{WBLUP_{MIX}}$) or *unweighted* ($\mathrm{mWBLUP_{MIX}}$) in the computation of the genomic estimated breeding values. The potential of training population devoid of race-specific resistant lines was furthermore tested by removing the respective lines based on mapped marker-trait associations ($\mathrm{GBLUP_{QDR(MTA)}}$) or with a disease severity smaller than x = 10%, x = 20%, x = 30%, x = 40%, or x = 50% ($\mathrm{GBLUP_{QDR(CULL_x)}}$).

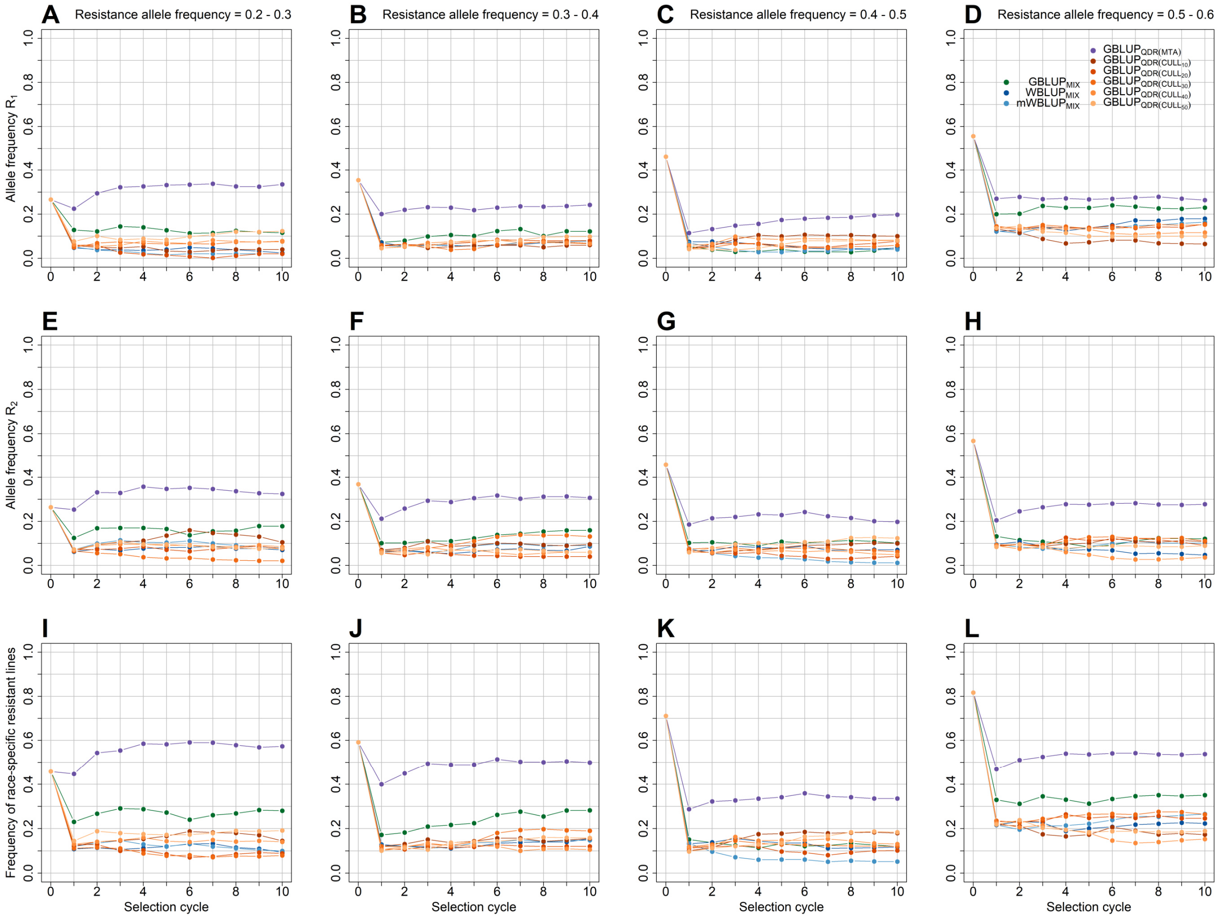

Figure S12 Frequency of the resistant allele R1 (A-D) and R2 (E-H) at the simulated race-specific R-genes as well as the frequency of race-specific resistant lines (I-L) in the recurrent selection when pre-selecting the sets of potential parents for the absence of race-specific R-gene mediated resistance (- R-gene strategy). Result are shown for a resistance allele frequency of 0.2-0.3 (A+E+I), 0.3-0.4 (B+F+J), 0.4-0.5 (C+G+K), and 0.5-0.6 (D+H+L) at the simulated R-genes in the founder population. It was assumed that the race-specific R-genes are still effective in the entire training population when fitting genomic best linear prediction models with a mixed training population of race-specific and race-nonspecific resistant genotypes ($\mathrm{GBLUP_{MIX}}$), which were compared with models including fixed effects for the most significant marker-trait associations, which were either *upweighted* ($\mathrm{WBLUP_{MIX}}$) or *unweighted* ($\mathrm{mWBLUP_{MIX}}$) in the computation of the genomic estimated breeding values. The potential of training population devoid of race-specific resistant lines was furthermore tested by removing the respective lines based on mapped marker-trait associations ($\mathrm{GBLUP_{QDR(MTA)}}$) or with a disease severity smaller than x = 10%, x = 20%, x = 30%, x = 40%, or x = 50% ($\mathrm{GBLUP_{QDR(CULL_x)}}$).

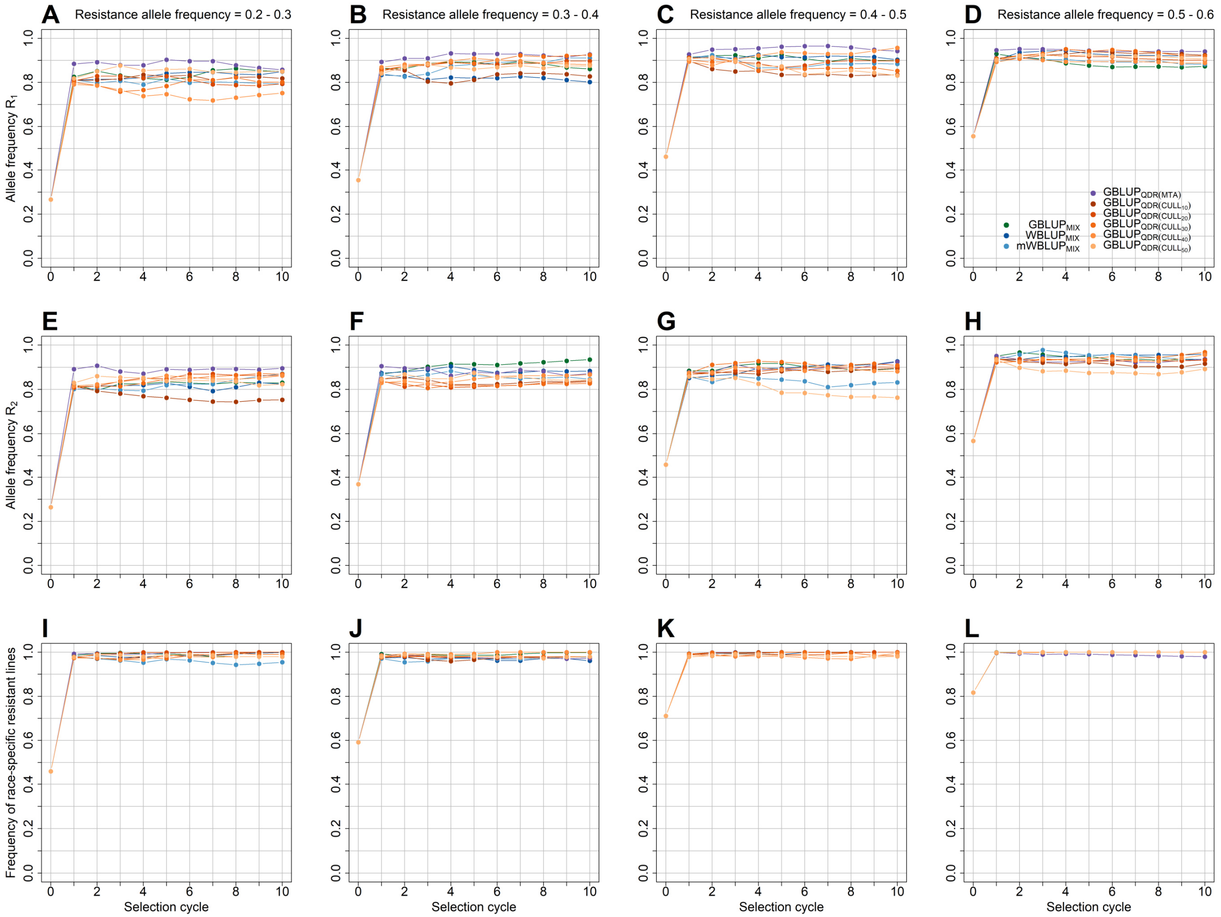

Figure S13 Frequency of the resistant allele R1 (A-D) and R2 (E-H) at the simulated race-specific R-genes as well as the frequency of race-specific resistant lines (I-L) in the recurrent selection when pre-selecting the sets of potential parents for the presence of race-specific R-gene mediated resistance (+ R-gene strategy). Result are shown for a resistance allele frequency of 0.2-0.3 (A+E+I), 0.3-0.4 (B+F+J), 0.4-0.5 (C+G+K), and 0.5-0.6 (D+H+L) at the simulated R-genes in the founder population. It was assumed that the race-specific R-genes are still effective in the entire training population when fitting genomic best linear prediction models with a mixed training population of race-specific and race-nonspecific resistant genotypes ($\mathrm{GBLUP_{MIX}}$), which were compared with models including fixed effects for the most significant marker-trait associations, which were either *upweighted* ($\mathrm{WBLUP_{MIX}}$) or *unweighted* ($\mathrm{mWBLUP_{MIX}}$) in the computation of the genomic estimated breeding values. The potential of training population devoid of race-specific resistant lines was furthermore tested by removing the respective lines based on mapped marker-trait associations ($\mathrm{GBLUP_{QDR(MTA)}}$) or with a disease severity smaller than x = 10%, x = 20%, x = 30%, x = 40%, or x = 50% ($\mathrm{GBLUP_{QDR(CULL_x)}}$).

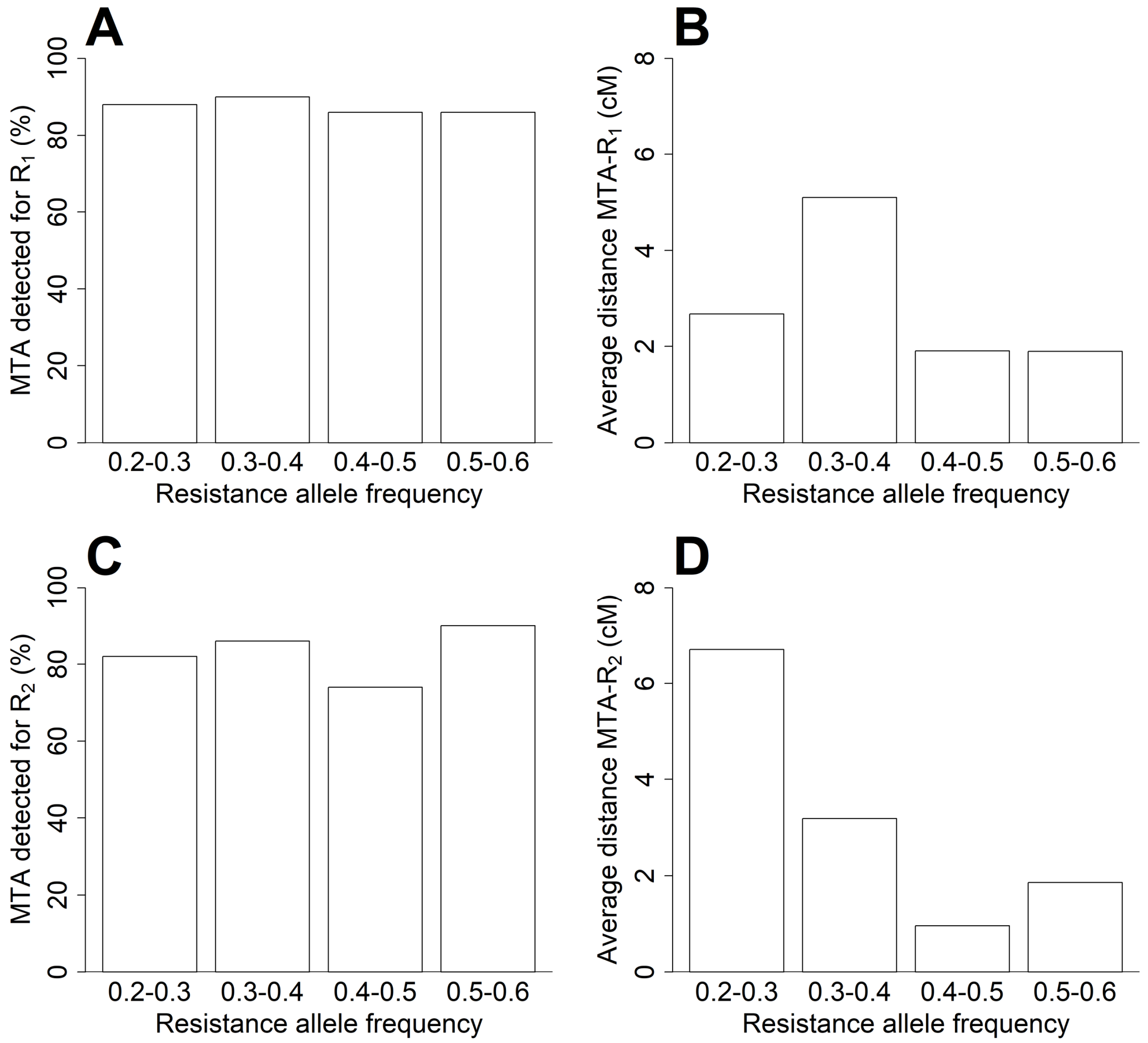

Figure S14 Averaged percentage of most significant marker-trait association (MTA) detected on the same chromosome as the simulated race-specific resistance genes R_1 and R_2 (A+C) by genome-wide association mapping in the training populations as well as the average genetic distance between the marker-trait associations and the causal variants (B+D).

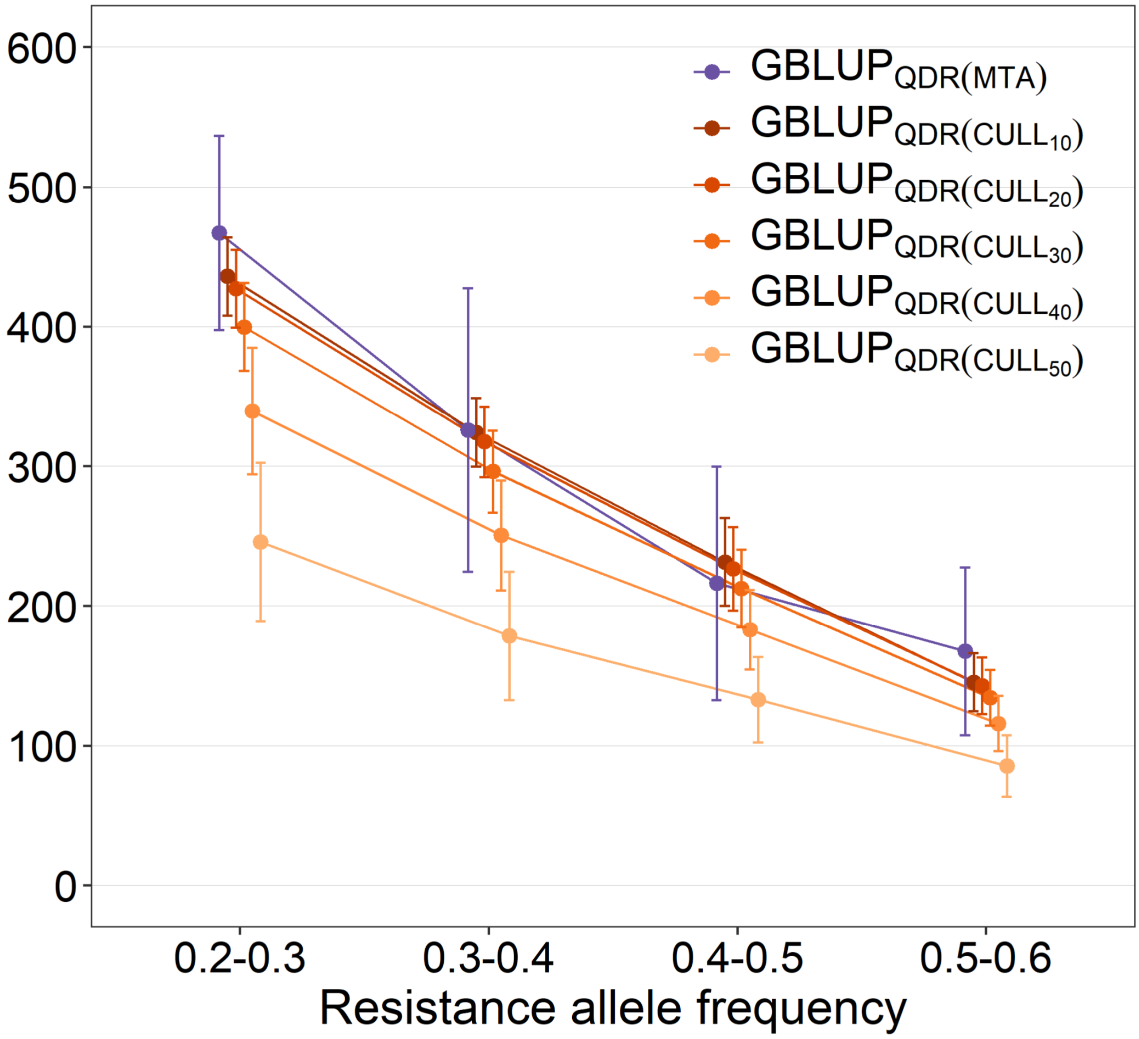

Figure S15 Average size of training populations (±standard error) devoid of race-specific resistant lines after removing the respective lines based on mapped marker-trait associations (GBLUP$_{QDR(MTA)}$) or with a disease severity smaller than 10-50% (GBLUP$_{QDR(CULL)}$). The training population size for the genomic best linear predictions (GBLUP$_{MIX}$) with a mixed training population of race-specific and race-nonspecific resistant genotypes and for models that included fixed effect for the most significant marker-trait associations, which were either *upweighted* (WBLUP$_{MIX}$) or *unweighted* (mWBLUP$_{MIX}$) in the computation of the genomic estimated breeding values was always set to 800 lines.
